# Application of Weighted Gene Co-expression Network Analysis for Data from Paired Design

**DOI:** 10.1038/s41598-017-18705-z

**Published:** 2018-01-12

**Authors:** Jianqiang Li, Doudou Zhou, Weiliang Qiu, Yuliang Shi, Ji-Jiang Yang, Shi Chen, Qing Wang, Hui Pan

**Affiliations:** 1 Faculty of Information Technology, Beijing University of Technology, Beijing, 100124 China; 2 Beijing Engineering Research Center for IoT Software and Systems, Beijing, 100124 China; 3 Channing Division of Network Medicine, Brigham and Women’s Hospital/Harvard Medical School, 181 Longwood Avenue, Boston, MA 02115 USA; 4 Tsinghua National Laboratory for Information Science and Technology, Tsinghua University, Beijing, 100084 China; 5 Department of Endocrinology, Peking Union Medical College Hospital, Chinese Academe of Medical Sciences & Peking Union Medical College, Beijing, 100730 China

## Abstract

Investigating how genes jointly affect complex human diseases is important, yet challenging. The network approach (e.g., weighted gene co-expression network analysis (WGCNA)) is a powerful tool. However, genomic data usually contain substantial batch effects, which could mask true genomic signals. Paired design is a powerful tool that can reduce batch effects. However, it is currently unclear how to appropriately apply WGCNA to genomic data from paired design. In this paper, we modified the current WGCNA pipeline to analyse high-throughput genomic data from paired design. We illustrated the modified WGCNA pipeline by analysing the miRNA dataset provided by Shiah *et al*. (2014), which contains forty oral squamous cell carcinoma (OSCC) specimens and their matched non-tumourous epithelial counterparts. OSCC is the sixth most common cancer worldwide. The modified WGCNA pipeline identified two sets of novel miRNAs associated with OSCC, in addition to the existing miRNAs reported by Shiah *et al*. (2014). Thus, this work will be of great interest to readers of various scientific disciplines, in particular, genetic and genomic scientists as well as medical scientists working on cancer.

## Introduction

Genetics plays an important role in the aetiologies of many complex human diseases. Genes are functional units of genetic materials. It is believed that whether a gene is expressed or not affects the synthesis of downstream proteins, which are the building blocks of the human body. However, recent studies have shown that individual genes do not work alone. Instead, genes interact with each other and jointly affect human health. Studies have shown that each gene is estimated on average to interact with four to eight other genes^1^ and to be involved in 10 biological functions^2^. Gene networks provide the potential to identify hundreds of genes that are associated with complex human diseases and that could serve as points for therapeutic interventions^3,4^, and this information is important for predicting the functions of new genes and finding genes that play key roles in complex human diseases. Constructing a gene co-expression network (GCN) is an effective way to characterize the correlation patterns among genes. Densely connected sub-networks form gene modules, which are usually related to biological functions. A gene co-expression network is an undirected graph, where each node corresponds to a gene, and each edge connects a pair of genes that are significantly correlated^5^.

Weighted gene co-expression network analysis (WGCNA)^6^ is a popular systems biology method used to not only construct gene networks but also detect gene modules and identify the central players (i.e., hub genes) within modules. The WGCNA pipeline is as follows: 1. Construct a gene co-expression network represented mathematically by an adjacency matrix, the element of which indicates co-expression similarity between a pair of genes. 2. Identify modules: WGCNA uses hierarchical clustering to identify modules. To measure the dissimilarity between clusters, WGCNA uses a topological overlap measure that can result in biologically meaningful modules in real data analysis. 3. Relate modules to phenotypes: several methods can be used to measure the association of a module to a phenotypic trait. For instance, one can test the association between the module eigengene (ME) and the phenotypic trait. The ME of a module is defined as the first principal component of the module. One can also use the module significance (MS), which is defined as the average gene significance (GS) of all genes in the module, to assess the association of a module to a phenotype. The GS of a node is defined as the correlation between the node and the phenotypic trait. Modules with high trait significance may represent pathways associated with the phenotypic trait. 4. Study inter-module relationships: WGCNA uses ME as a representative profile of a module and quantifies module similarity by eigengene correlation. Studying the relationship of the modules can help to find which modules are highly related. 5. Find key drivers in interesting modules: the nodes having the largest number of edges are most important because the malfunction of this gene would affect all connected genes. WGCNA assumes that genetic networks obey the scale-free topology criterion. Instead of dichotomizing gene co-expression (connected = 1, unconnected = 0), WGCNA uses a ‘soft’ threshold to determine the weights of the edges connecting pairs of genes, which has been proven to yield more robust results than unweighted networks^7^. An appropriate soft threshold will make the resulting co-expression network closer to a scale-free network. Instead of relating individual genes to phenotype, WGCNA focuses on the relationship between a few modules and the trait, which greatly alleviates the multiple testing problem inherent in microarray data analysis^8^. WGCNA is widely used in genomic data analysis, in which samples are assumed independent of each other.

**Figure 1:**
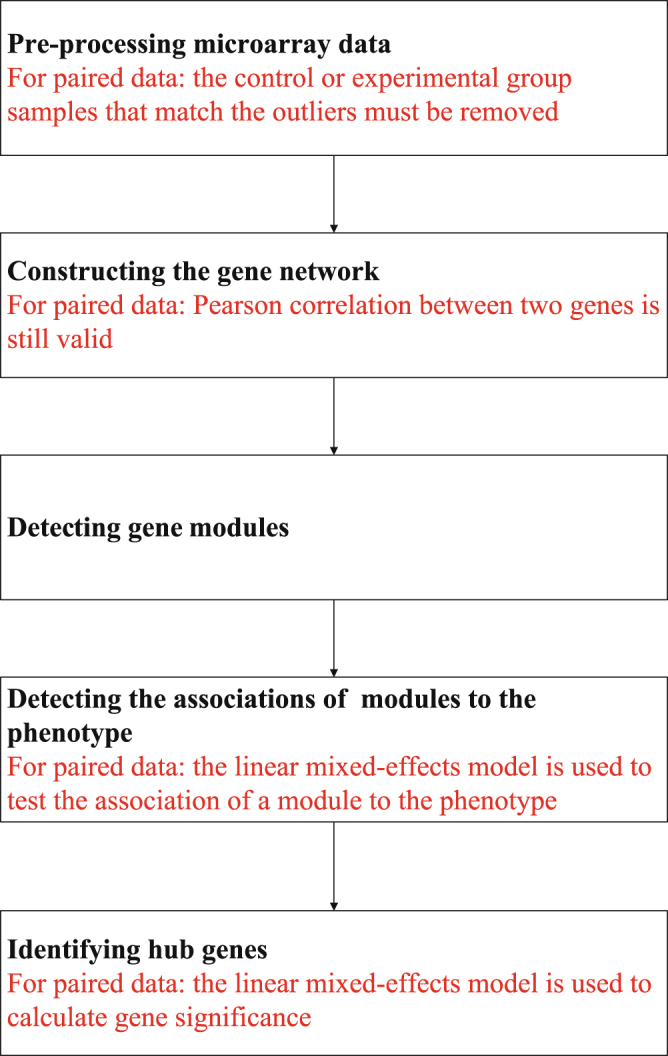
Flow chart of the modified WGCNA pipeline.

The paired design is powerful for reducing the confounding effects and has been used successfully in genomic studies. However, it is currently unclear how to appropriately apply WGCNA to genomic data from paired design. For independent data, WGCNA uses the Pearson correlation to measure the magnitude of co-expression between nodes (such as a gene or microRNA) in a network. Can we use the Pearson correlation to measure the magnitude of co-expression between two nodes for paired samples? How do we evaluate the associations of gene modules to the phenotype of interest for data from paired design? How do we calculate gene significance for data from paired design? To address these question, we propose in this article to modify the current WGCNA pipeline. The modified pipeline can be divided into five steps, as is shown in Fig. 1. We illustrate the modified pipeline using the Gene Expression Omnibus (GEO)^9,10^ dataset (GSE45238)^11^ to investigate the associations of microRNAs to oral squamous cell carcinomas (OSCC).

## Results

**Figure 2:**
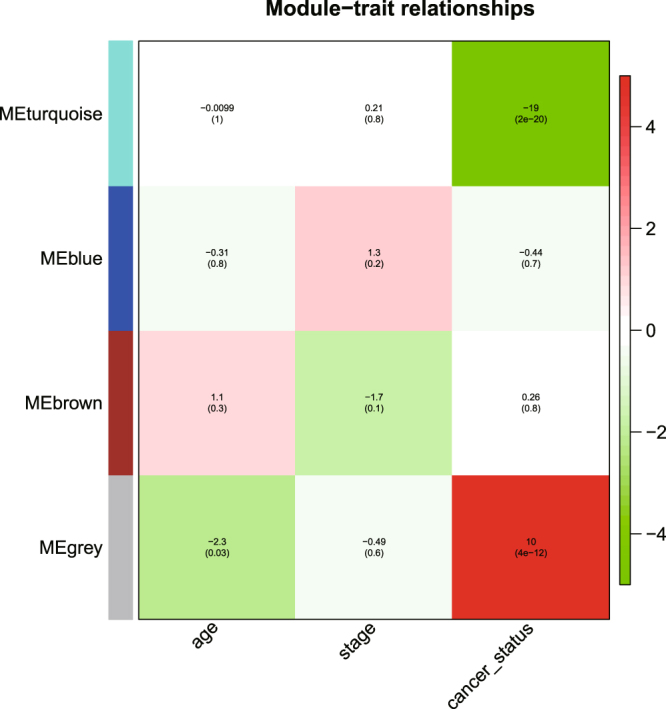
Module-trait association. Each row corresponds to a module; each column corresponds to a trait. Each cell contains the test statistic value and its corresponding p value from the linear mixed-effects model.

**Figure 3:**
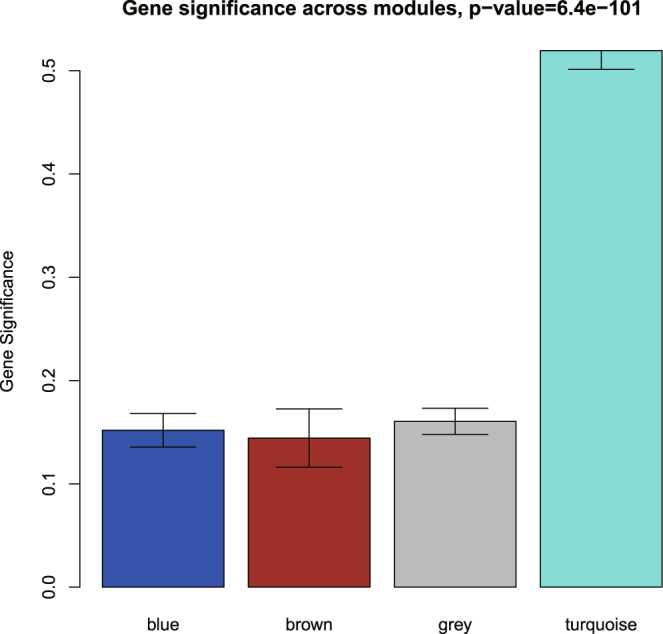
Barplot of module significance defined as the mean gene significance across all genes in the module.

In this work, we showed that (1) the Pearson correlation could be used to measure the magnitude of co-expression between two genes regardless of whether the samples are paired or independent and (2) to evaluate the associations of modules/genes to phenotypes, we need to account for the within-pair correlation by appropriate statistical models, such as the linear mixed effects model (LMM). Based on the WGCNA pipeline we modified, we identified four miRNA modules (Supplementary Fig. 9) for the OSCC data. There were 254 miRNAs in the turquoise module, 189 miRNAs in the blue module, 78 miRNAs in the brown module, and 309 miRNAs in the grey module. Some MEs are highly correlated based on the hierarchical clustering analysis (Supplementary Fig. 10). The result of the linear mixed-effects model shows that the turquoise module (t-value = −18.68, p-value = 1.97e-20) and the grey module (t-value = 10, p-value = 4e-12) are significantly associated with cancer status (see Fig. 2). We also compared the MS among the modules (see Fig. 3), and the results showed that the turquoise module had the highest relevance to cancer status.

**Figure 4:**
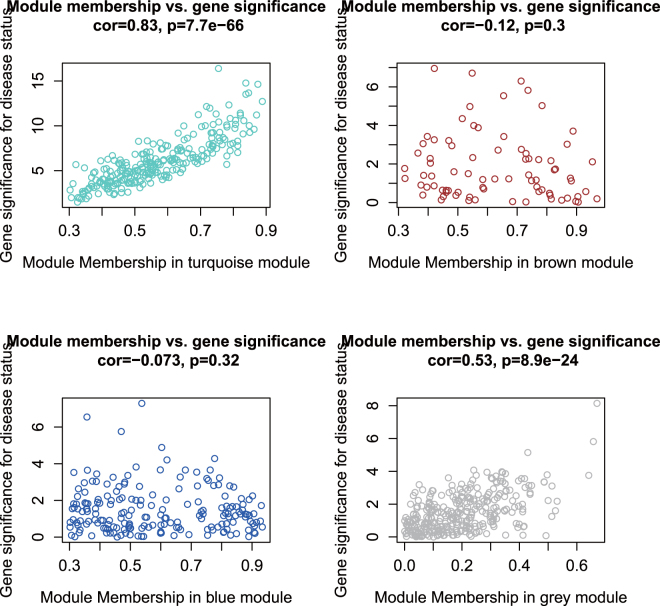
Correlation between MM and GS of all miRNAs in each module.

For each miRNA in a module, we drew the module membership (MM) against the GS in a scatter plot (see Fig. 4). The module membership (MM): *MM*(*i*) = *cor*(*x*_*i*_, *ME*) is defined in WGCNA to measure the importance of the gene within the module. The greater the absolute value of *MM*(*i*) is, the more important the gene *i* is in the module^6^. It can be seen that the GS in the turquoise module is highly correlated with MM, illustrating that miRNAs significantly associated with cancer status are often also the important elements of the turquoise module.

**Figure 5:**
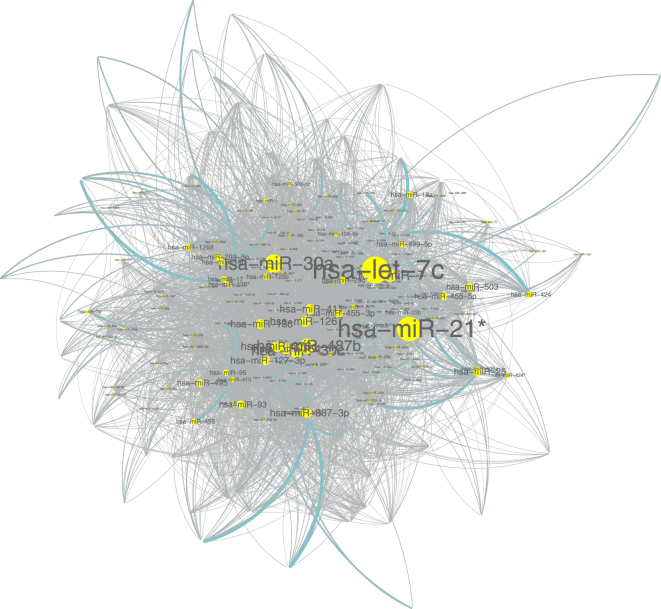
Network of miR-let-7 in the turquoise module.

For the turquoise module, the hub is miR-let-7c, which is connected to 140 miRNAs (see Fig. 5). According to Wikipedia (https://en.wikipedia.org/wiki/Let-7_microRNA_precursor), let-7 acts as a tumour suppressor. Manikandan *et al*.^12^ showed that let7-a, let-7d, and let-7f are differentially expressed in OSCC. Hui AB *et al*.^13^ showed that let-7c was down-regulated in head and neck squamous cell carcinoma (HNSCC). The turquoise module contains the two miRNAs (miR-329 and miR-410) that were identified by Shiah *et al*.^11^. The information about miR-let-7c, miR-329 and miR-410 is listed in Table 1. Seventy-one of the 84 miRNAs detected by Shiah *et al*. are in the turquoise module and none of them are in the blue or brown modules.

**Table 1:** Network properties of miR-let-7c, miR-329 and miR-410.

miRNA	Gene Significance	Module Membership	Degree	Module
miR-let-7c	0.84	0.87	140	turquoise
miR-329	0.41	0.59	45	turquoise
miR-410	0.64	0.79	100	turquoise

We uploaded the miRNAs in the turquoise module and the grey module to miRsystem^14^ and obtained 9233 targets in the turquoise module and 8629 targets in the grey module. There were 7628 overlapping targets. The results showed that the top 6 enriched KEGG pathways for the two modules are the same: Pathways in Cancer, mapk Signalling Pathway, Axon Guidance, Wnt signalling pathway, neurotrophin signalling pathway and focal adhesion. The 6 enriched KEGG pathways have all been previously reported to be related to OSCC^11,15–18^.

## Discussion

WGCNA is widely used in genomic data analysis, in which samples are independent of each other. In this paper, we modified the current WGCNA pipeline to analyse high-throughput genomic data from paired design. We demonstrated that it is feasible to construct co-expression networks using the Pearson correlation for paired data. To relate modules to phenotype, we used a linear mixed-effects model to account for the within-pair correlations. We calculated the gene significance of a node as the absolute value of the test statistic of the linear mixed effects model for testing the association of the node to the phenotype. We analysed the miRNA expression profile data (GSE45238) from a paired design study contributed to GEO by Shiah *et al*.^11^ to illustrate the utility of the modified pipeline.

In this real data analysis, we identified one co-expression miRNA subnetwork (turquoise module) that was significantly associated with OSCC status and a set of OSCC-associated miRNAs (the grey module) that are not co-expressed among each other. The result of miRsystem showed that the top 6 enriched KEGG pathways for the two modules are the same and have all been previously reported to be related to OSCC^11,15–18^. The turquoise module (with 254 miRNAs) contains most of the 84 miRNAs identified by the probe-wise approach in Shiah *et al*.^11^. None of those 84 miRNAs are in the blue and brown modules that are not associated with OSCC. This is assuring and indicates that (1) most of the 84 miRNAs are in a co-expressed network and (2) more OSCC-related miRNAs could be found by using network approaches than by using the probe-wise approach.

In summary, the real data analysis showed that the modified WGCNA pipeline could identify biologically relevant modules. The limitations of our study include the small sample size and the lack of independent data to replicate our results. Further investigation is warranted.

This article demonstrated that miRNAs can form network modules that regulate human genes. It will be interesting to further investigate if the two detected miRNA modules may intertwine with other cellular networks. For instance, Tibiche and Wang (2008)^19^ showed that miRNAs preferentially regulate hub nodes and cut points of the network of metabolites, while avoiding regulating intermediate nodes.

It will also be interesting to investigate if miRNAs can be used to predict clinical outcomes, such as the risk of developing OSCC. It has been reported that highly connected network genes (i.e., hub genes) can correctly classify tumour subtypes^20^. To obtain better prediction performance, we can use both network hubs and network motifs, such as a positive regulatory loop^21^. To obtain robust (i.e., reproducible) prediction results, we can incorporate human signalling networks^22^, protein interaction networks^23^ and gene ontology information^24^ into the prediction process.

## Methods

### OSCC and microRNA

Oral squamous cell carcinomas (OSCC) is the sixth most common cancer worldwide. OSCC is the cause of over 400,000 cancer-related deaths each year, with 80 percent of deaths occurring in developing countries^25^. Despite the progress made in OSCC treatment, the survival rate of patients after 5 years has not significantly improved due to late diagnosis, frequent loco-regional recurrences at the primary site and metastatic neck lymph nodes after treatment^15,26^. MicroRNAs (miRNAs) are a kind of endogenous small length (22 nt) RNAs that have many important regulatory effects in the cell. Studies have shown that miRNAs are involved in the growth, differentiation, apoptosis, invasion, and metastasis of OSCC tumour cells^27^. Shiah *et al*.^11^ conducted a global microarray analysis of miRNA and detected eighty-four miRNAs differentially expressed in the OSCC specimens compared with the matched tissue. By using qRT-PCR and RT-PCR, these authors predicted two miRNAs, miR329 and miR410, that could potentially target Wnt-7b, an activator of the Wnt-b-catenin pathway, thereby attenuating the Wnt-b-catenin signalling pathway in OSCC. Importantly, the dysregulation of the Meg-3-miR329 and -410-Wnt-7b-b-catenin signalling axis may result from exposure to betel quid chewing. However, how miRNAs interplay with each other to contribute to the development of OSCC is still largely unknown. In this paper, we used WGCNA to detect OSCC-associated miRNA subnetworks (modules) based on the expression data of OSCC investigated by Shiah *et al*.^11^.

### Data

The data used in this paper was obtained from the GEO database in NCBI (Gene Expression Omnibus^9,10^, http://www.ncbi.nlm.nih.gov/geo), and the platform data entry number is GPL8179. The experimental data entry number is GSE45238. The dataset comes from the work of Shiah S *et al*.^11^ in 2013. Forty OSCC specimens and their matched non-tumourous epithelial counterparts were selected in the dataset. There were 858 miRNAs in the dataset; we kept 830 miRNAs from Target Mature Version 12 for further analysis.

### Constructing gene network

In the co-expression network of genes, nodes are genes, and edges indicate the magnitude of their co-expression. The variable *x*_*i*_ is denoted as the expression profile of the i-th gene, and WGCNA calculates the co-expression of genes based on an adjacency matrix. WGCNA defines the adjacency matrix based on co-expression similarity *s*_*ij*_ between the i-th gene and the j-th gene. By default, *s*_*ij*_ is defined as the absolute value of the Pearson correlation coefficient between the profiles of genes *i* and *j*^6^:

1 $${s}_{ij}=|cor({x}_{i},{x}_{j})|$$

In statistics, the Pearson correlation is used to measure the linear correlation of two random variables. The Pearson correlation is also called inter-class correlation. Correspondingly, there is a concept called intra-class correlation in statistics, which was proposed to modify inter-class correlation to handle the case of paired measurements. It is natural to think that we should use intra-class correlation to measure the correlation between two genes when expression data are collected from paired design, in which the two samples within a pair are correlated. However, the intra-class correlation is not appropriate for measuring the correlation between two genes since it is used for repeated measurements of the same random variable in two correlated samples, not for two random variables (i.e., two genes). In this article, we showed that the Pearson correlation can be used to measure the magnitude of the co-expression of a pair of genes regardless of whether the data are from independent design or from paired design (See Supplementary Text). We showed

2 $$\begin{array}{c}corr({Z}_{1},{Z}_{2})=\frac{Cov({Z}_{1},{Z}_{2})}{\sqrt{Var({Z}_{1})Var({Z}_{2})}}\\ \quad \quad \quad \quad \quad =\,\frac{\mathrm{(1}-p)Cov({X}_{1},{X}_{2})+pCov({Y}_{1},{Y}_{2})+p\mathrm{(1}-p){\delta }_{1}{\delta }_{2}}{\sqrt{[\mathrm{(1}-p){\sigma }_{{X}_{1}}^{2}+p{\sigma }_{{Y}_{1}}^{2}+p\mathrm{(1}-p){\delta }_{1}^{2}]\,[\mathrm{(1}-p){\sigma }_{{X}_{2}}^{2}+p{\sigma }_{{Y}_{2}}^{2}+p\mathrm{(1}-p){\delta }_{2}^{2}]}}\mathrm{.}\end{array}$$

In Equation (2), ${Z}_{i}=\mathrm{(1}-\theta ){X}_{i}+\theta {Y}_{i}$ represents the expression level for the i-th gene, where *θ* indicates if the sample is from a case (*θ* = 1) or from a control (*θ* = 0), *X*_*i*_ is the expression level of the i-th gene for control tissue samples, and *Y*_*i*_ is the expression level of the i-th gene for tumour tissue samples. Equation (2) is true regardless of whether samples are paired or not, so the Pearson correlation between two genes is still valid for paired samples. Next, the co-expression similarity *s*_*ij*_ is transformed into the adjacency *a*_*ij*_ via an adjacency function^28^:

3 $${a}_{ij}={s}_{ij}^{\beta }$$

where $\beta \ge 1$ is a soft threshold and is determined according to a scale-free topology criterion since the literature shows that most of the biological networks have the scale-free topology.

### Detecting gene modules

A gene module is a cluster of densely interconnected genes in terms of co-expression. WGCNA uses hierarchical clustering to identify gene modules and colour to indicate modules. For genes that are not assigned to any of the modules, WGCNA places them in a grey module. That is, genes in the grey module are not co-expressed. The module eigengene (ME) of a module is defined as the first principal component of the module and represents the overall expression level of the module.

### Detecting associations of modules to phenotype

To account for the within-pair correlation in data from paired design, the linear mixed-effects model (LMM) is used to test the association of a module to phenotype. In the OSCC data analysis, we used the following model for testing the association of a miRNA module to the tumour status (normal vs tumour):

4 $${y}_{ij}={\beta }_{0i}+{\beta }_{1}\cdot tumou{r}_{j}+{\beta }_{2}\cdot ag{e}_{i}+{\beta }_{3}\cdot stag{e}_{i}+{e}_{ij}\quad i=1,\ldots ,n,\quad j=1,\,2$$

In the above model, *y*_*ij*_ is the expression level of the eigengene of the i-th subject for the j-th tissue sample. *j* = 1 indicates the control sample, and *j* = 2 indicates the tumour sample. *tumour*_1_ = 0 indicates the control tissue, and *tumour*_2_ = 1 indicates the tumour tissue. *age*_*i*_ is the age for the i-th subject. *stage*_*i*_ is the tumor stage for the i-th subject. *β*_0*i*_ is the subject-specific random intercept to account for the within-pair correlation, and *e*_*ij*_ is the random error term. We assume ${\beta }_{{\rm{0}}i}\sim N({\beta }_{{\rm{0}}},{\tau }^{2})$, ${e}_{ij}\sim N\mathrm{(0},\,{\sigma }^{2})$, and *β*_*0i*_ and *e*_*ij*_ are mutually independent.

Another way to assess the association of a module to a phenotype in WGCNA is with the module significance (MS), which is defined as the average gene significance (GS) of all genes in the module. For data from paired design, the GS of a miRNA is defined as the absolute value of the test statistic of the linear mixed effects model for testing the association of the miRNA to the tumour status. By comparing the MS between modules, we can identify the modules that are highly related to the phenotype^6^.

### Applying WGCNA to the OSCC data

We first used the R Bioconductor packages iCheck and lumi to draw the quantile plot and the scatter plot of principal components to check whether there were outlying probes, samples/arrays, and/or batch effects (Supplementary Figs 1 and 2). After data preprocessing, we repeated the principal component analysis to double check the data quality. No outlying probes were detected (Supplementary Fig. 3). We also observed that tumour samples and normal samples are separated in the PCA plot (Supplementary Fig. 4). Then, we performed hierarchical clustering on the samples to further detect potential outliers. We identified 2 outliers in the control group. We excluded these 2 control arrays and corresponding 2 matched tumour samples. The remaining 76 samples were used for further analysis (Supplementary Fig. 5). Next, we used R package WGCNA to perform the weighted correlation network analysis. We chose the soft threshold *β* = 7 to construct the co-expression network as the *R*^2^ reached the peak for the first time when *β* = 7 (Supplementary Fig. 6). The plot of *log*10(*p*(*k*)) versus *log*10(*k*) (Supplementary Fig. 7) indicates that by using *β* = 7, the network is close to a scale-free network, where k is the whole network connectivity and p(k) is the corresponding frequency distribution. When *β* = 7, the *R*^2^ is 0.98, ensuring that the network was close to the scale-free network. After the soft thresholding power *β* was determined, the Topological Overlap Matrix (TOM) (Supplementary Fig. 8) and *dissTOM* = 1−*TOM* were obtained. A hierarchical clustering of MEs was performed to study the correlations among the modules. To account for the within-pair correlation in data from paired design, we used the linear mixed-effects model (equation (4)) for testing the association of a module to the tumor status. We visualized the network that consists of the hub miR-let-7 and its connected nodes in the turquoise module by using Cytoscape^29^, which is an open source software platform that is primarily used to visualize molecular interactions and biological pathways. In this study, we regarded a miRNA as the hub of a module if its degree (i.e., the number of edges) is the largest among all miRNAs in the module. For the grey module, we did not try to find a hub since miRNAs in this module are not co-expressed. To understand how miRNAs participate in the regulation of gene expression during pathogenic processes, it is useful to predict target genes and perform KEGG pathway enrichment analysis. In this study, the web tool miRsystem was used to predict the genes targeted by miRNAs. MiRsystem has integrated several prediction algorithms and experimentally validated data sources to reduce false positive predictions^14^.

### Data availability

The datasets generated and/or analysed during the current study are available in the Gene Expression Omnibus (GEO) repository, https://www.ncbi.nlm.nih.gov/geo/query/acc.cgi?acc=GSE45238.

## Electronic supplementary material

Supplementary Information
