# Supplementary material for: Application of Weighted Gene Co-expression Network Analysis for Data from Paired Design
Source: Sci Rep. 2018 Jan 12;8:622. doi: 10.1038/s41598-017-18705-z (PMC5766625; doi:10.1038/s41598-017-18705-z)
Supplement: Supplementary file 1 — Supplementary Information [file 41598_2017_18705_MOESM1_ESM.pdf]

Supplementary Information for

Application of Weighted Gene Co-expression Network Analysis for Data from Paired Design

Jianqiang Li[1,2], Doudou Zhou[1,+], Weiliang Qiu[3], Yuliang Shi[1,2], Ji-Jiang Yang[4,*], Shi Chen[5], Qing Wang[4], Hui Pan[5]

Affiliations:

[1]Faculty of Information Technology, Beijing University of Technology, Beijing, 100124, China

[2] Beijing Engineering Research Center for IoT Software and Systems, Beijing, 100124, China

[3] Channing Division of Network Medicine, Brigham and Women's Hospital/Harvard Medical School, 181 Longwood Avenue, Boston MA 02115, USA

[4] Tsinghua National Laboratory for Information Science and Technology, Tsinghua University, Beijing, 100084, China

[5] Department of Endocrinology, Peking Union Medical College Hospital, Chinese Academe of Medical Sciences & Peking Union Medical College, Beijing, 100730, China

*Corresponding author. Email: jijiangyang@126.com

[+]Co-first author

Contents

Supplementary text: Correlation between two microRNAs for paired data

Supplementary Figure 1: Quantile plot for probes from Target Mature Version 12 before log2 transformation and quantile normalization.

Supplementary Figure 2: Plot of pcas for probes from Target Mature Version 12 before log2 transformation and quantile normalization.

Supplementary Figure 3: Quantile plot for probes from Target Mature Version 12 after log2 transformation and quantile normalization.

Supplementary Figure 4: Plot of pcas for probes from Target Mature Version 12 after log2 transformation and quantile normalization.

Supplementary Figure 5: Remove the outliers.

Supplementary Figure 6: Selecting soft-thresholding power.

Supplementary Figure 7: The test of property of scale-free network.

Supplementary Figure 8: Heatmap plot of miRNA network.

Supplementary Figure 9: Clustering dendrogram of miRNAs, with dissimilarity based on topological overlap.

Supplementary Figure 10: Visualization of the relationship among module eigengenes and cancer status.

Supplementary Table 1: List of the 254 miRNAs in the turquoise module.

Supplementary Table 2: List of the 309 miRNAs in the grey module.

Supplementary Text. Correlation between two microRNAs for paired data.

Suppose we would like to calculate the Pearson correlation between two microRNAs (m_1 and m_2) for n pairs of tissue samples. Denote the random variable X_1 as the gene expression of the microRNA m_1 for control tissue samples. Denote the random variable Y_1 as the gene expression of the microRNA m_1 for tumor tissue samples. Denote the random variable X_2 as the gene expression of the microRNA m_2 for control tissue samples. Denote the random variable Y_2 as the gene expression of the microRNA m_2 for tumor tissue samples.

Let θ be the indicator indicating if a sample is from a tumor tissue or from a control tissue. We assume θ follows a Bernoulli distribution with parameter p. Then

$$\mathrm{E}(\theta) = p = \Pr(\theta = 1) = \Pr\text{ (a sample is from a tumor tissue)} \tag{1}$$

Then we can represent the gene expression level for the microRNA m_1 as

$$Z_1 = (1-\theta)X_1 + \theta Y_1 \tag{2}$$

That is, given $\theta = 1$, then $Z_1 = Y_1$. Given $\theta = 0$, then $Z_1 = X_1$.

Similarly, we can represent the gene expression level for the microRNA m_2 as

$$Z_2 = (1-\theta)X_2 + \theta Y_2 \tag{3}$$

That is, given $\theta = 1$, then $Z_2 = Y_2$. Given $\theta = 0$, then $Z_2 = X_2$.

Denote

$$\begin{aligned} \delta_1 &= E(Y_1) - E(X_1), \\ \delta_2 &= E(Y_2) - E(X_2) \end{aligned} \tag{4}$$

We assume that θ is independent of X_1, Y_1, X_2 and Y_2. We then have

$$\begin{aligned} \mathrm{E}(Z_1) &= E(1-\theta)E(X_1) + E(\theta)E(Y_1) \\ &= (1-p)\mu_1 + p(\mu_1 + \delta_1) \\ &= \mu_1 + p\delta_1 \\ \mathrm{E}(Z_2) &= (1-p)\mu_2 + p(\mu_2 + \delta_2) \\ &= \mu_2 + p\delta_2 \end{aligned} \tag{5}$$

By definition of variance, we have

$$\mathrm{Var}(Z_1) = E[Z_1^2] - \{E[Z_1]\}^2 \tag{6}$$

We can get

$$E[Z_1^2] = E[(1-\theta)X_1 + \theta Y_1]^2$$
$$= E[(1-\theta)^2 X_1^2 + 2(1-\theta)\theta X_1 Y_1 + \theta^2 Y_1^2]$$
$$\xlongequal{(1-\theta)^2 = (1-\theta),\ \theta^2 = \theta} E[(1-\theta)X_1^2 + (\theta-\theta)X_1 Y_1 + \theta Y_1^2]$$
$$\xlongequal{\theta \text{ and } X_1 \text{ and } X_2 \text{ are independent}} (1-p)E(X_1^2) + pE(Y_1^2) \tag{7}$$
$$= (1-p)\{Var(X_1) + [E(X_1)]^2\} + p\{Var(Y_1) + [E(Y_1)]^2\}$$
$$= (1-p)[\sigma_{X_1}^2 + \mu_1^2] + p[\sigma_{Y_1}^2 + (\mu_1 + \delta_1)^2]$$
$$= (1-p)\sigma_{X_1}^2 + p\sigma_{Y_1}^2 + (1-p)\mu_1^2 + p(\mu_1 + \delta_1)^2$$

And we can get

$$\begin{aligned}[E(Z_1)]^2 &= [(1-p)\mu_1 + p(\mu_1) + \delta_1]^2 \\ &= (1-p)^2\mu_1^2 + p^2(\mu_1+\delta_1)^2 + 2p(1-p)\mu_1(\mu_1+\delta_1)\end{aligned} \tag{8}$$

Hence, we have

$$\begin{aligned}Var(Z_1) &= (1-p)\sigma_{X_1}^2 + p\sigma_{Y_1}^2 + (1-p)\mu_1^2 + p(\mu_1+\delta_1)^2 \\ &\quad -[(1-p)^2\mu_1^2 + p^2(\mu_1+\delta_1)^2 + 2p(1-p)\mu_1(\mu_1+\delta_1)] \\ &= (1-p)\sigma_{X_1}^2 + p\sigma_{Y_1}^2 + p(1-p)\mu_1^2 + p(1-p)(\mu_1+\delta_1)^2 - 2p(1-p)\mu_1(\mu_1+\delta_1) \\ &= (1-p)\sigma_{X_1}^2 + p\sigma_{Y_1}^2 + p(1-p)[\mu_1 - (\mu_1+\delta_1)^2] \\ &= (1-p)\sigma_{X_1}^2 + p\sigma_{Y_1}^2 + p(1-p)\delta_1^2\end{aligned} \tag{9}$$

Similarly, we can get

$$\text{Var}(Z_2) = (1-p)\sigma_{X_2}^2 + p\sigma_{Y_2}^2 + p(1-p)\delta_2^2 \tag{10}$$

By definition, the covariance between Z_1 and Z_2 is as follows

$$\text{Cov}(Z_1, Z_2) = E[Z_1 Z_2] - E(Z_1)E(Z_2) \tag{11}$$

We can get

$$
\begin{aligned}
E(Z_1Z_2) &= E\{[(1-\theta)X_1 + \theta Y_1][(1-\theta)X_2 + \theta Y_2]\} \\
&= E\{(1-\theta)^2 X_1X_2 + (1-\theta)\theta X_1Y_2 + \theta(1-\theta)Y_1X_2 + \theta^2 Y_1Y_2\} \\
&= E\{(1-\theta)X_1X_2 + \theta Y_1Y_2\} \\
&= (1-p)E(X_1X_2) + pE(Y_1Y_2) \\
&= (1-p)[Cov(X_1,X_2) + E(X_1)E(X_2)] + p[Cov(Y_1,Y_2) + E(Y_1)E(Y_2)] \\
&= (1-p)[Cov(X_1,X_2) + \mu_1\mu_2] + p[Cov(Y_1,Y_2) + (\mu_1+\delta_1)(\mu_2+\delta_2)] \\
&= (1-p)Cov(X_1,X_2) + pCov(Y_1,Y_2) \\
&\quad + (1-p)\mu_1\mu_2 + p\mu_1\mu_2 + p\mu_1\delta_2 + p\mu_2\delta_1 + p\delta_1\delta_2 \\
&= (1-p)Cov(X_1,X_2) + pCov(Y_1,Y_2) + \mu_1\mu_2 + p\mu_1\delta_2 + p\mu_2\delta_1 + p\delta_1\delta_2
\end{aligned} \tag{12}
$$

We also can get

$$
\begin{aligned}
E(Z_1)E(Z_2) &= (\mu_1 + p\delta_1)(\mu_2 + p\delta_2) \\
&= \mu_1\mu_2 + p\mu_1\delta_2 + p\mu_2\delta_1 + p^2\delta_1\delta_2
\end{aligned} \tag{13}
$$

Therefore, we can get

$$
\begin{aligned}
Cov(Z_1,Z_2) &= (1-p)Cov(X_1,X_2) + pCov(Y_1,Y_2) + \mu_1\mu_2 + p\mu_1\delta_2 + p\mu_2\delta_1 + p\delta_1\delta_2 \\
&\quad - (\mu_1\mu_2 + p\mu_1\delta_2 + p\mu_2\delta_1 + p^2\delta_1\delta_2) \\
&= (1-p)Cov(X_1,X_2) + pCov(Y_1,Y_2) + p(1-p)\delta_1\delta_2
\end{aligned} \tag{14}
$$

Thus, the correlation between Z_1 and Z_2 is

$$
\begin{aligned}
corr(Z_1,Z_2) &= \frac{Cov(Z_1,Z_2)}{\sqrt{Var(Z_1)Var(Z_2)}} \\
&= \frac{(1-p)Cov(X_1,X_2) + pCov(Y_1,Y_2) + p(1-p)\delta_1\delta_2}{\sqrt{[(1-p)\sigma_{X_1}^2 + p\sigma_{Y_1}^2 + p(1-p)\delta_1^2][(1-p)\sigma_{X_2}^2 + p\sigma_{Y_2}^2 + p(1-p)\delta_2^2]}}
\end{aligned} \tag{15}
$$

Note that Formula (7) is correct for both paired samples (i.e., X_1 and Y_1 are dependent) and independent samples (i.e., X_1 and Y_1 are independent). Hence, Formula (15) is true no matter whether samples are paired or not.

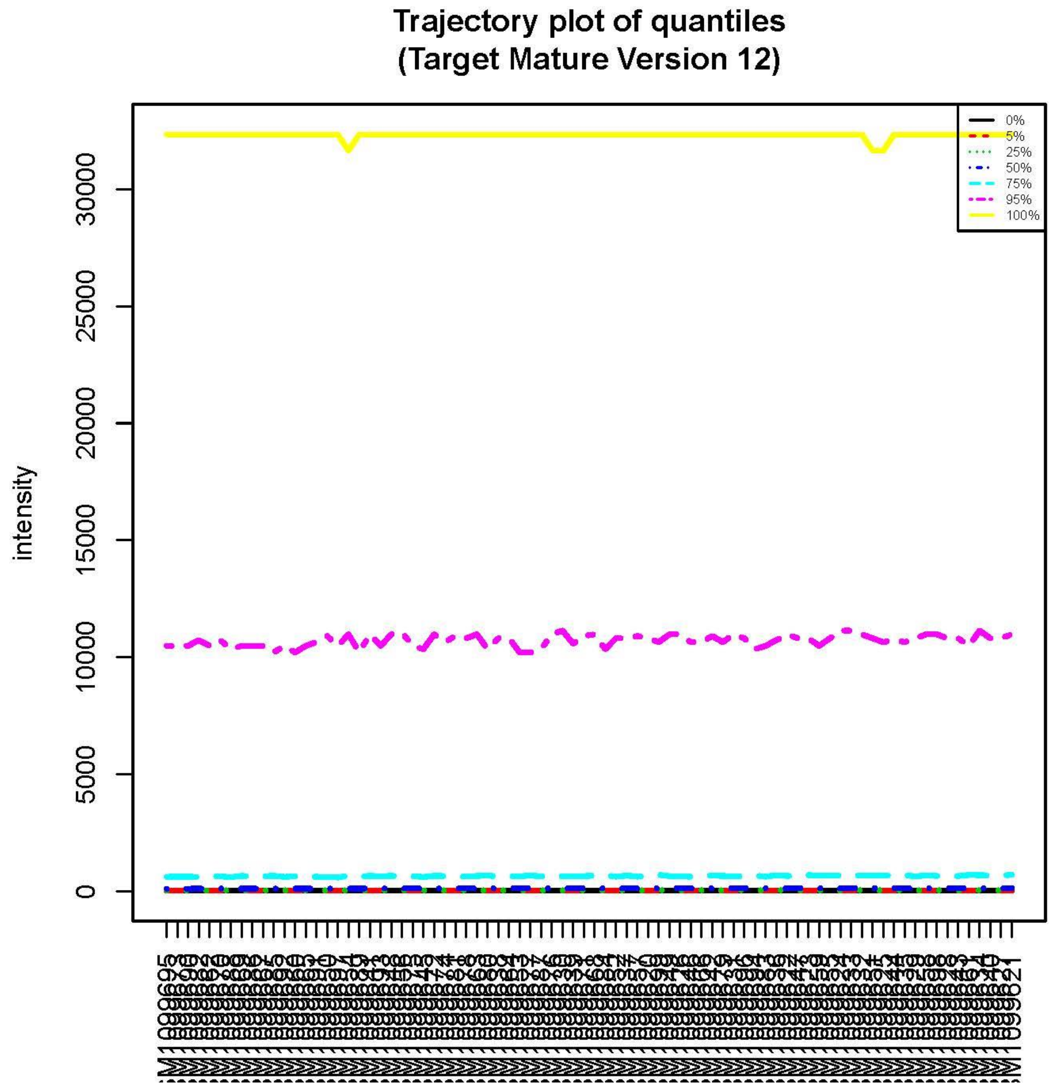

Supplementary Figure 1. Quantile plot for probes from Target Mature Version 12 before log2 transformation and quantile normalization.

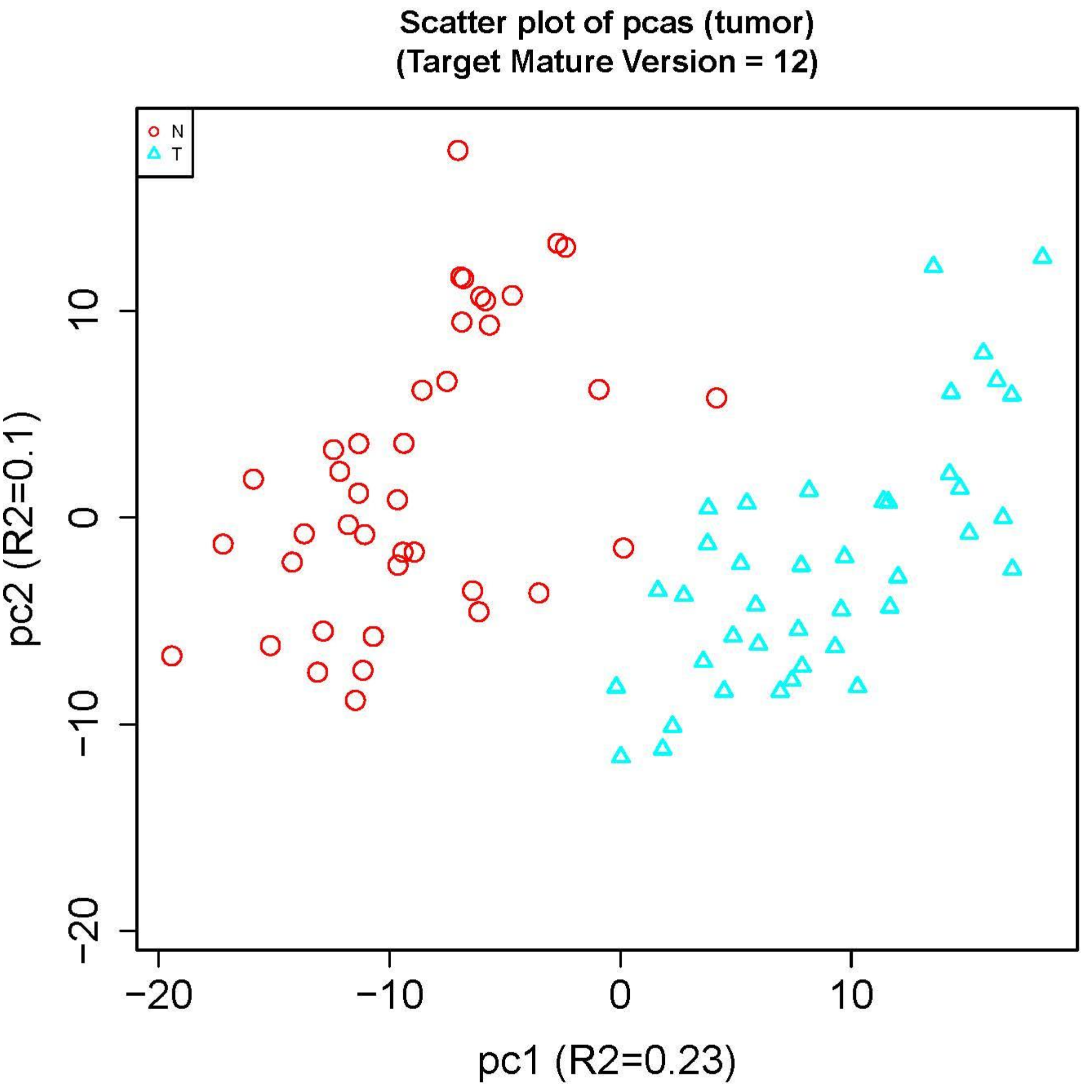

Supplementary Figure 2. Plot of pcas for probes from Target Mature Version 12 before log2 transformation and quantile normalization.

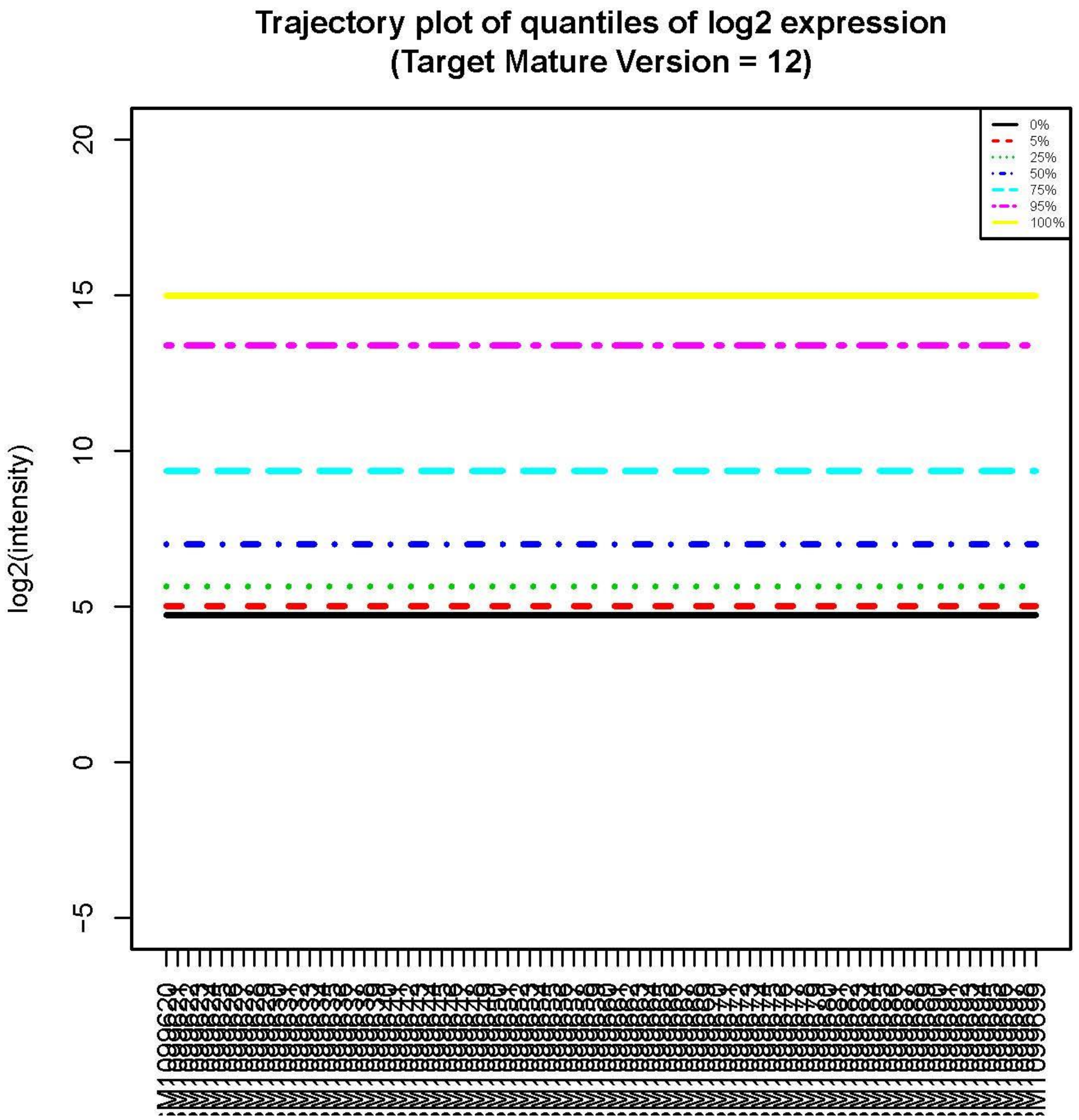

Supplementary Figure 3. Quantile plot for probes from Target Mature Version 12 after log2 transformation and quantile normalization.

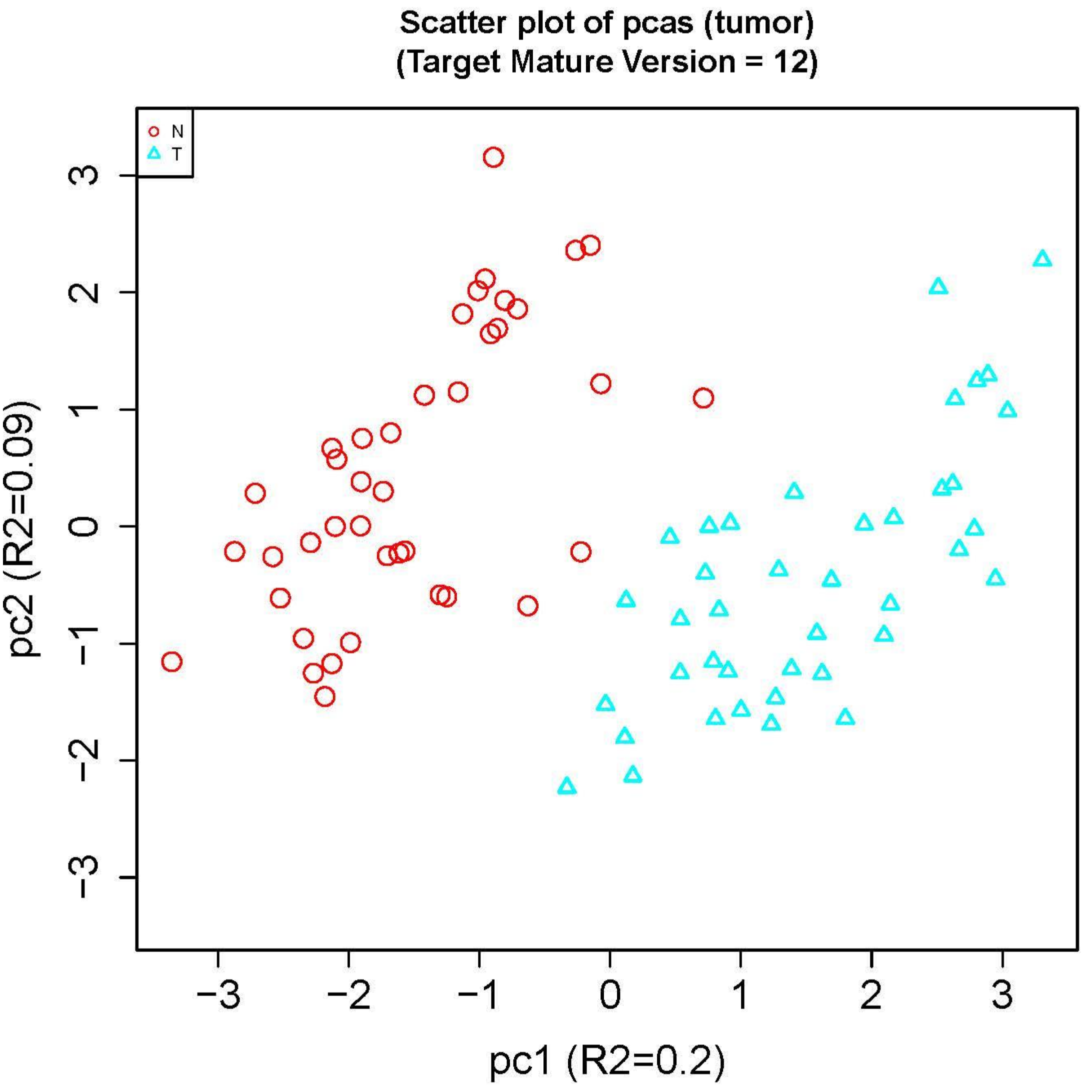

Supplementary Figure 4. Plot of pcas for probes from Target Mature Version 12 after log2 transformation and quantile normalization.

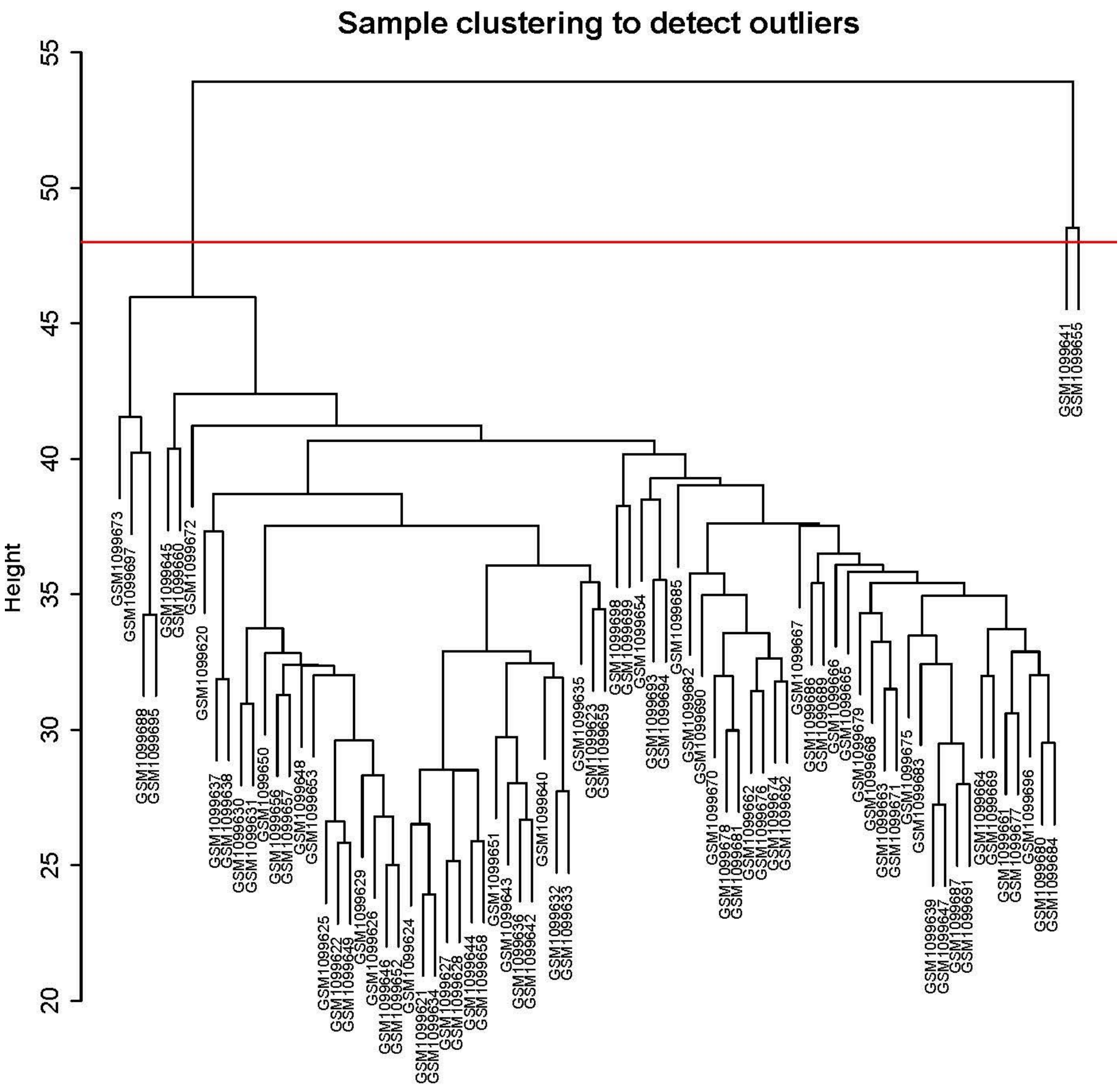

Supplementary Figure 5. Remove the outliers. The red line in the plot shows the height cut that removes the offending samples.

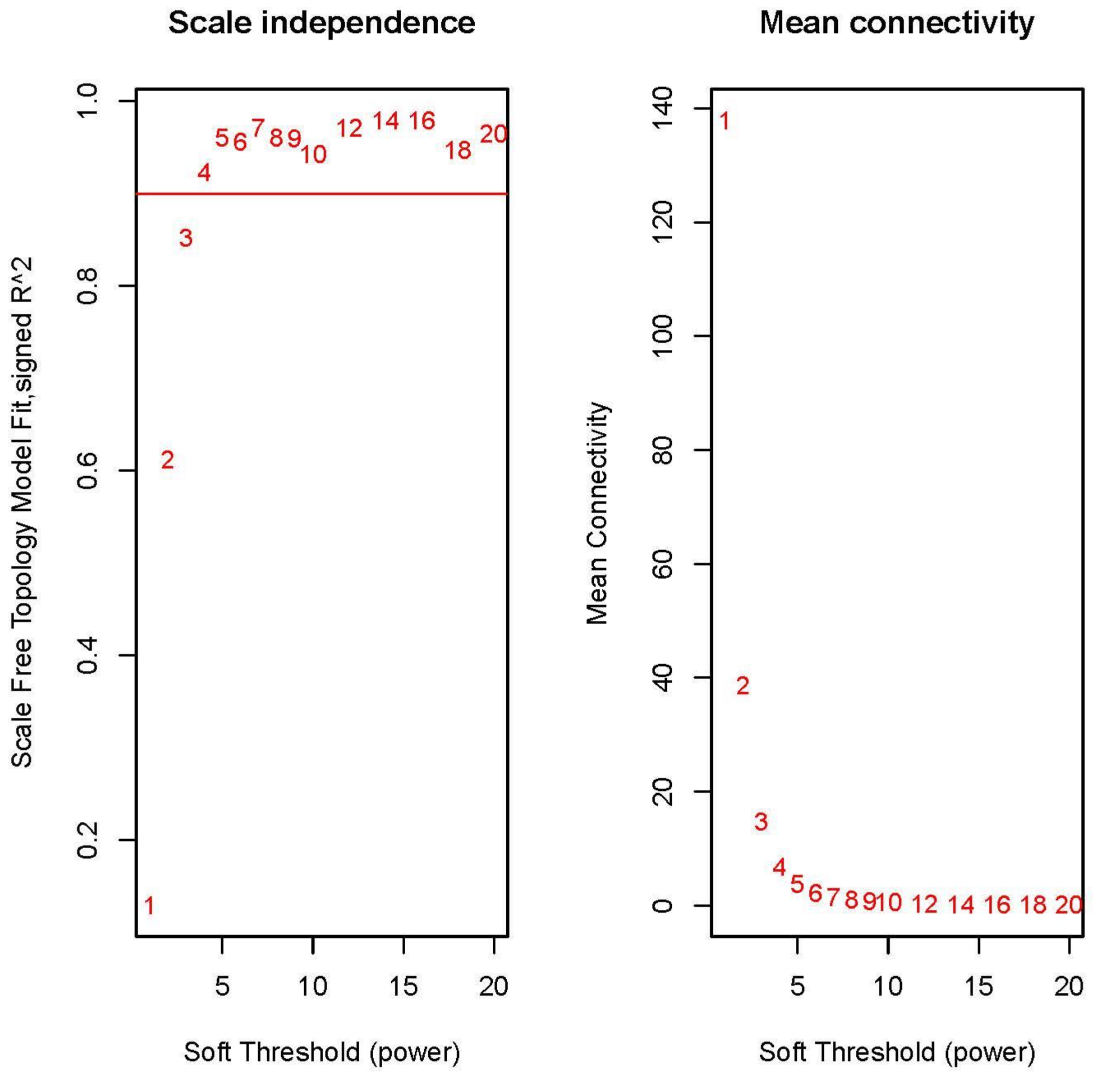

Supplementary Figure 6. Selecting soft-thresholding power. In the left panel, the vertical axis represents the square of the correlation between log (k) and log (p (k)), i.e. the model fitting index R^2 of the linear model that regresses log(p(k)) on log(k). The connectivity k_i of node i equals the number of its direct connections to other nodes. P(k) represents the frequency distribution of the connectivity. The higher the coefficient, the closer the network is to the distribution of the scale free network. The right panel displays the mean connectivity under different weighting coefficients.

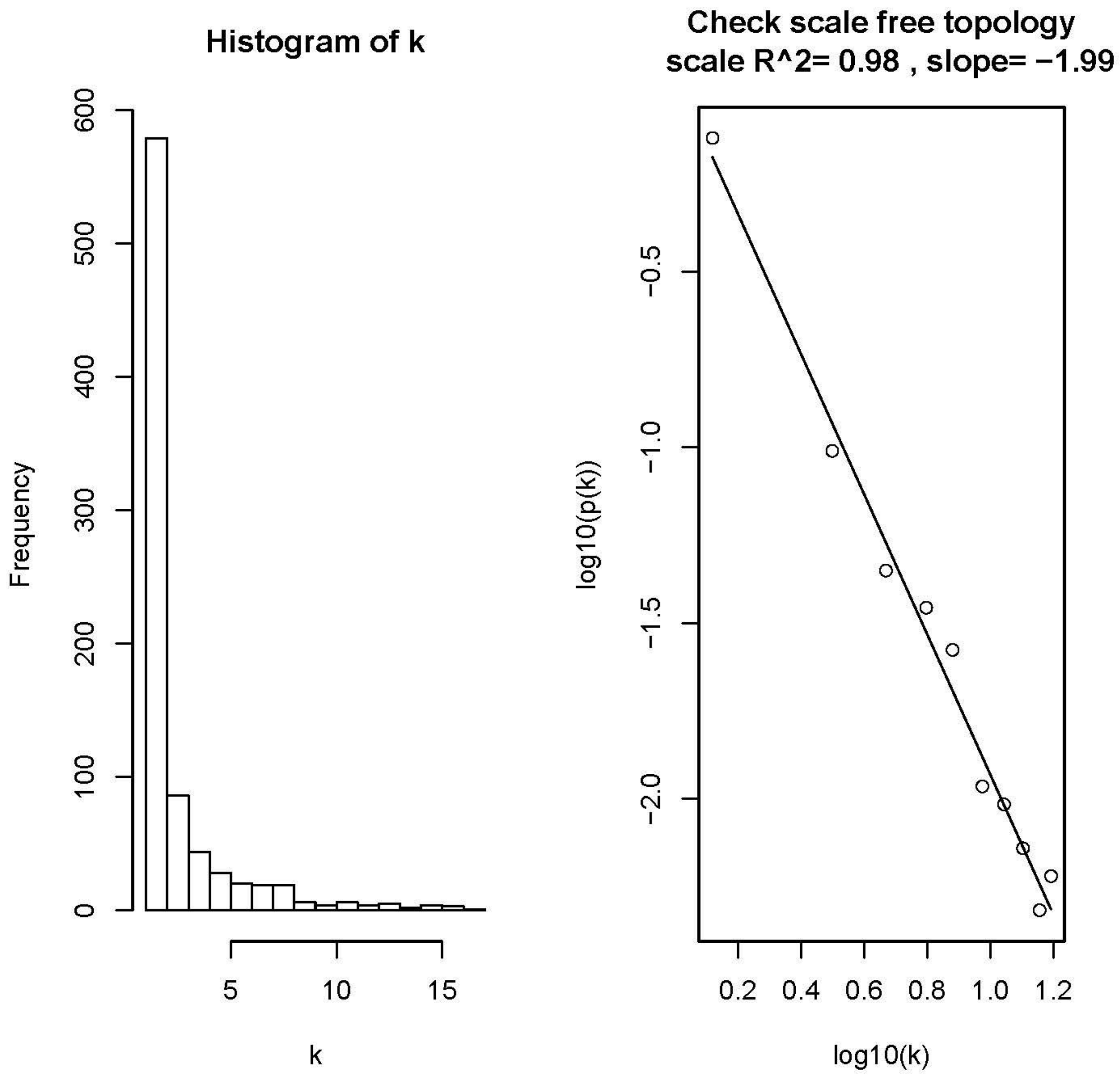

Supplementary Figure 7. The test of property of scale-free network. The left panel shows the distribution of the connectivity of each node in the network. The connectivity k_i of node i equals the number of its direct connections to other nodes. P(k) represents the frequency distribution of the connectivity. The right panel shows the scatter plot of log (p (k)) and log (k).

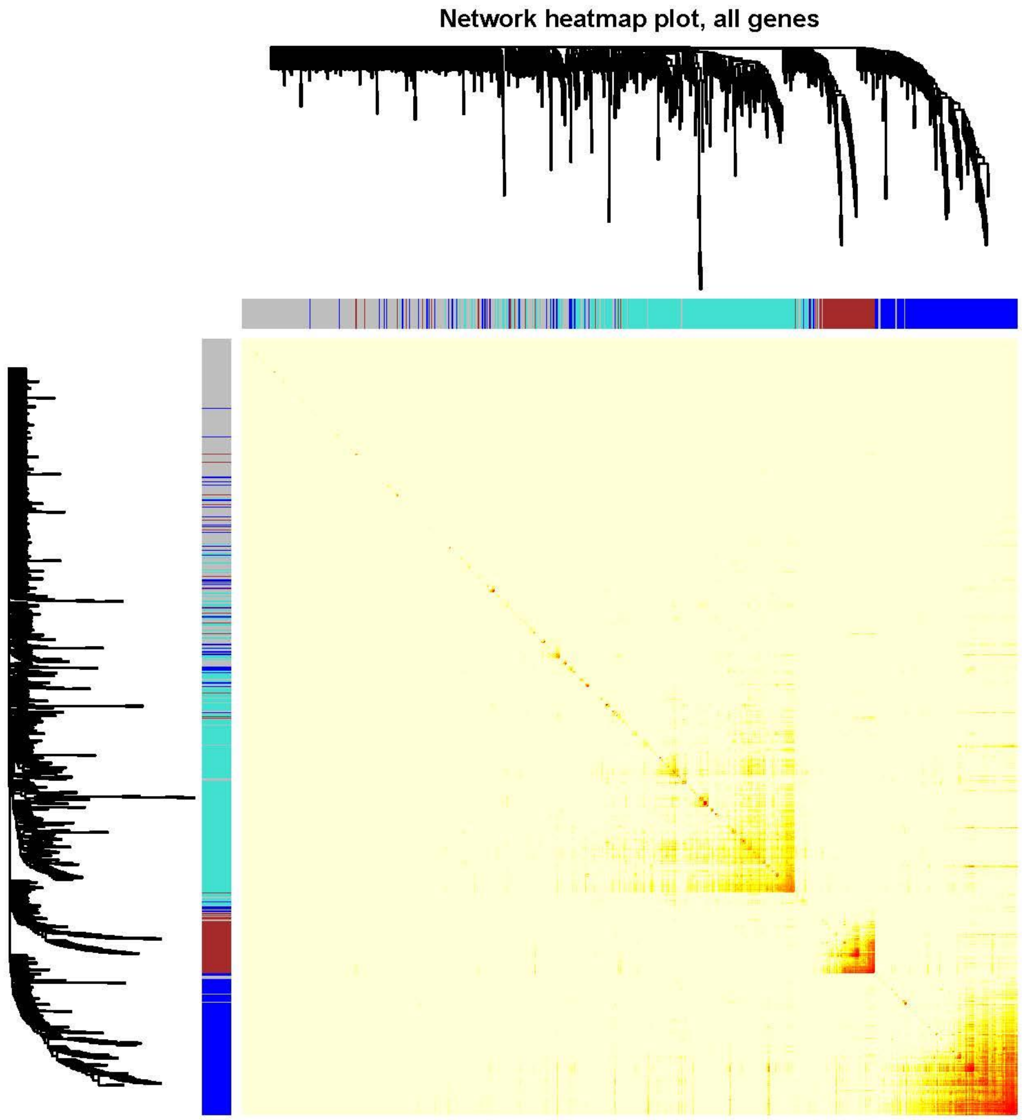

Supplementary Figure 8. Heatmap plot of miRNA network. The heatmap depicts the Topological Overlap Matrix (TOM) among all miRNAs in the analysis. Light color represents low overlap and progressively darker red color represents higher overlap.

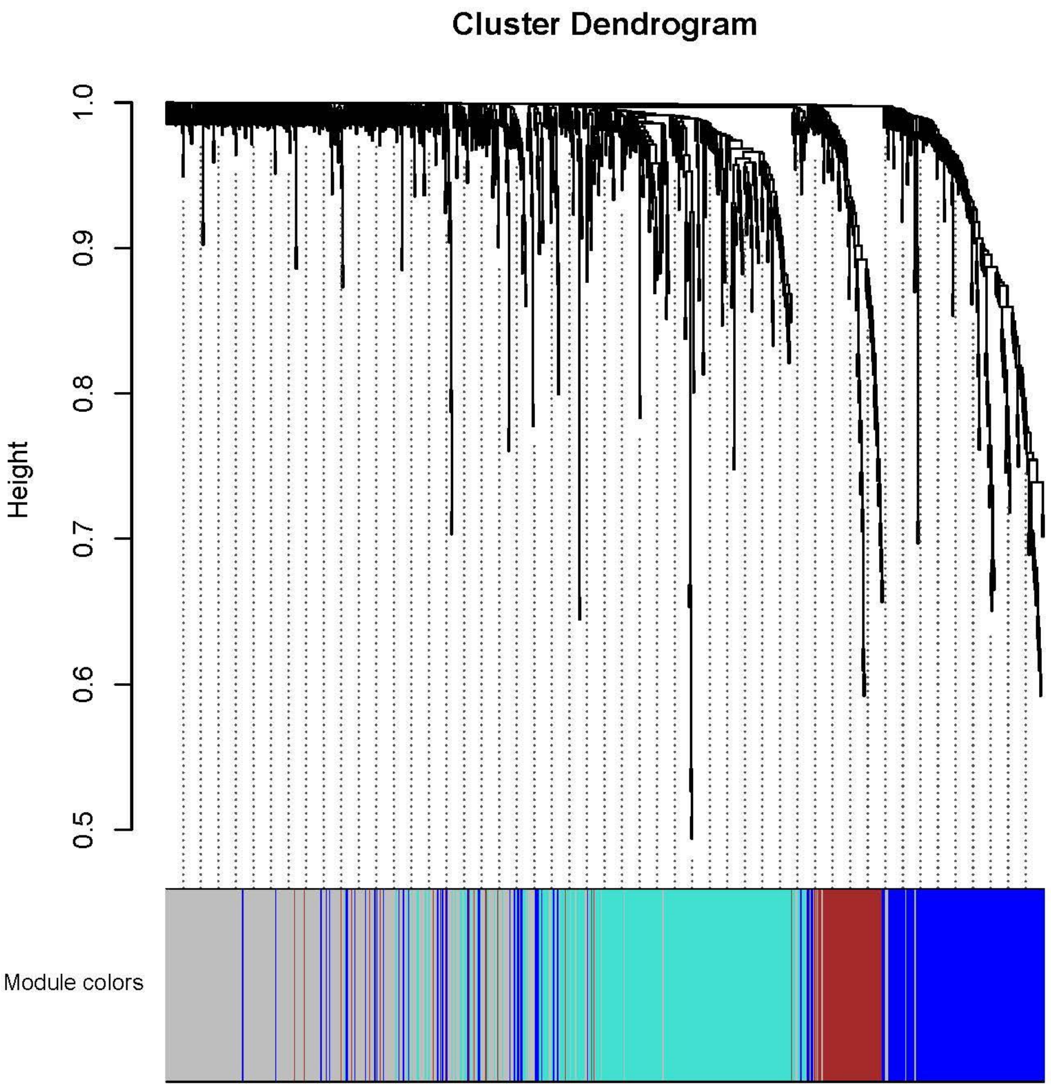

Supplementary Figure 9. Clustering dendrogram of miRNAs, with dissimilarity based on topological overlap. Different colors represent different modules. Three modules are identified. Grey bars represent miRNAs that do not belong to any other modules and are not co-expressed.

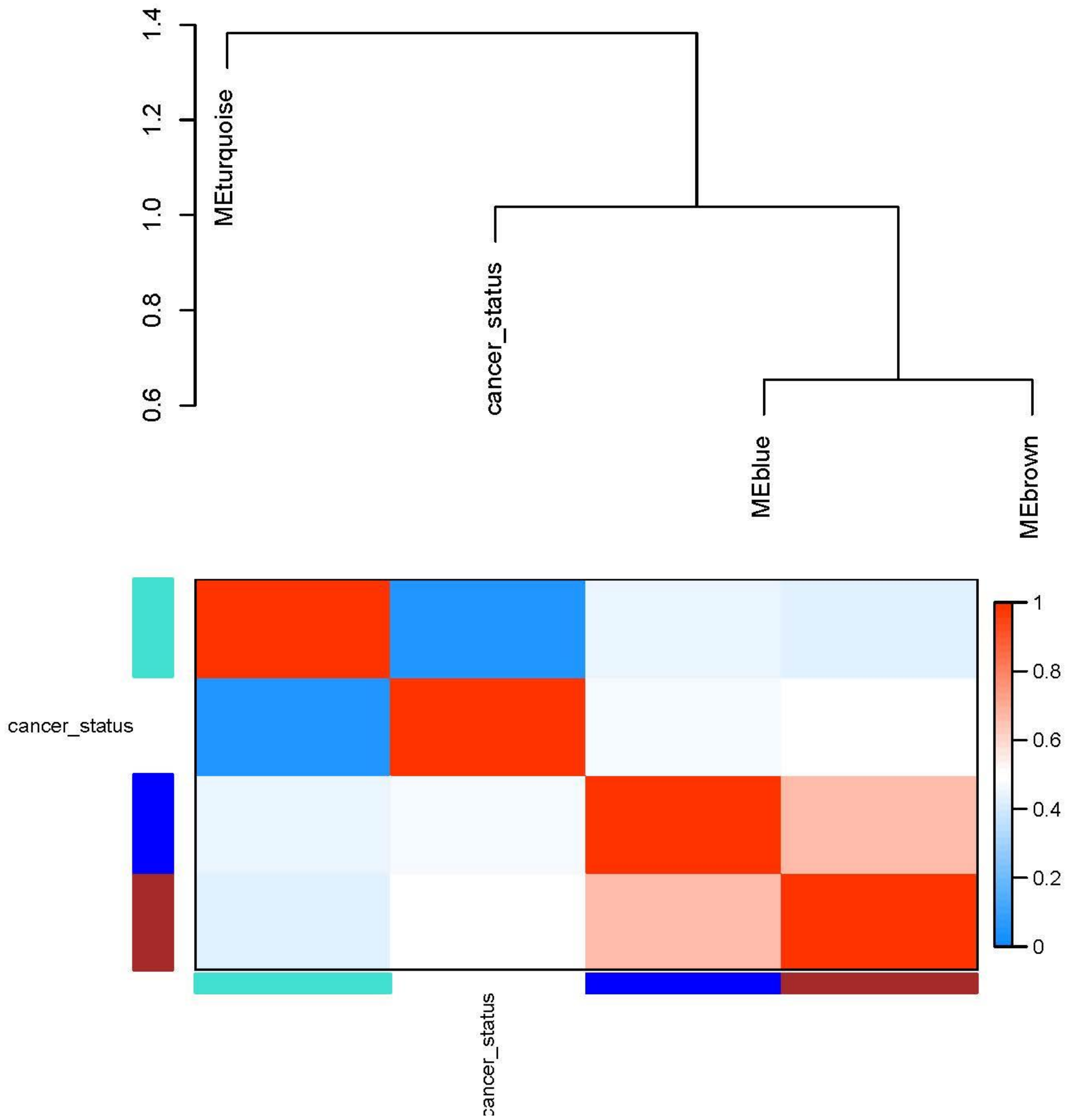

Supplementary Figure 10. Visualization of the relationship among module eigengenes and cancer status. The upper panel shows a hierarchical clustering dendrogram of the module eigengenes. The heatmap in the lower panel shows the eigengene adjacency.

Supplementary Table 1. List of the 254 miRNAs in the turquoise module.

miRNA	stat	pval	GS	MMturquoise
hsa-miR-329	-4.27	1.32E-04	4.27	0.59
hsa-miR-424	10.00	4.60E-12	10.00	-0.65
hsa-miR-376c	-9.62	1.30E-11	9.62	0.86
hsa-miR-487a	-1.92	6.29E-02	1.92	0.33
hsa-miR-202	-3.97	3.16E-04	3.97	0.38
hsa-miR-369-5p	-5.19	7.73E-06	5.19	0.65
hsa-miR-182*	3.64	8.33E-04	3.64	-0.42
hsa-miR-143	-4.52	6.17E-05	4.52	0.55
hsa-miR-95	7.93	1.69E-09	7.93	-0.73
hsa-miR-223	5.64	1.94E-06	5.64	-0.54
hsa-miR-92a	4.01	2.83E-04	4.01	-0.58
hsa-miR-33b	3.27	2.35E-03	3.27	-0.41
hsa-miR-139-5p	-10.83	5.04E-13	10.83	0.79
hsa-miR-340*	-2.49	1.75E-02	2.49	0.34
hsa-miR-153	-3.10	3.64E-03	3.10	0.52
hsa-miR-378	-7.59	4.74E-09	7.59	0.64
hsa-miR-548b-3p	-3.78	5.54E-04	3.78	0.49
hsa-miR-127-3p	-8.06	1.15E-09	8.06	0.80
hsa-miR-214	-4.40	8.82E-05	4.40	0.58
hsa-miR-495	-7.23	1.39E-08	7.23	0.79
hsa-miR-15b	7.91	1.81E-09	7.91	-0.77
hsa-miR-542-3p	6.11	4.49E-07	6.11	-0.40
hsa-miR-130b	6.16	3.84E-07	6.16	-0.44
hsa-miR-208b	-5.79	1.19E-06	5.79	0.65
hsa-miR-181a	5.85	9.93E-07	5.85	-0.45
hsa-miR-140-5p	-4.83	2.40E-05	4.83	0.53
hsa-miR-593*	4.72	3.34E-05	4.72	-0.54
hsa-miR-553	5.38	4.30E-06	5.38	-0.52
hsa-miR-30a*	-10.44	1.41E-12	10.44	0.76
hsa-miR-371-3p	4.68	3.79E-05	4.68	-0.48
hsa-miR-542-5p	8.33	5.15E-10	8.33	-0.54
hsa-miR-18a*	6.00	6.25E-07	6.00	-0.68
hsa-miR-195	-8.49	3.26E-10	8.49	0.77
hsa-miR-9*	-2.59	1.37E-02	2.59	0.42
hsa-miR-802	3.33	1.98E-03	3.33	-0.44
hsa-miR-502-5p	-9.22	4.00E-11	9.22	0.73
hsa-miR-370	-5.88	9.22E-07	5.88	0.66
hsa-miR-891a	-5.24	6.76E-06	5.24	0.41
hsa-miR-485-5p	-3.93	3.54E-04	3.93	0.52
hsa-miR-30d	-6.79	5.36E-08	6.79	0.68
hsa-miR-375	-9.88	6.35E-12	9.88	0.78
hsa-miR-382	-5.91	8.24E-07	5.91	0.64
hsa-miR-486-5p	-7.78	2.69E-09	7.78	0.60
hsa-miR-518b	3.80	5.23E-04	3.80	-0.50
hsa-miR-410	-7.95	1.59E-09	7.95	0.79
hsa-miR-592	-4.94	1.68E-05	4.94	0.48
hsa-miR-199b-5p	-3.62	8.75E-04	3.62	0.55
hsa-miR-19b	4.38	9.45E-05	4.38	-0.51
hsa-miR-557	4.02	2.77E-04	4.02	-0.41
hsa-miR-489	-3.62	8.75E-04	3.62	0.45
hsa-miR-10b	-4.86	2.16E-05	4.86	0.38
hsa-miR-34b*	9.22	3.98E-11	9.22	-0.73
hsa-miR-338-3p	-10.16	3.00E-12	10.16	0.74

hsa-miR-107	-6.21	3.29E-07	6.21	0.46
hsa-miR-92b	4.25	1.38E-04	4.25	-0.37
hsa-miR-362-5p	-3.54	1.09E-03	3.54	0.40
hsa-miR-490-5p	-1.50	1.41E-01	1.50	0.33
hsa-miR-21	6.34	2.20E-07	6.34	-0.56
hsa-miR-26b	-5.70	1.60E-06	5.70	0.55
hsa-miR-628-3p	-6.29	2.52E-07	6.29	0.62
hsa-miR-607	-5.03	1.30E-05	5.03	0.46
hsa-miR-497	-6.05	5.44E-07	6.05	0.70
hsa-miR-379	-7.40	8.44E-09	7.40	0.73
hsa-miR-30b	-6.85	4.46E-08	6.85	0.52
hsa-miR-617	-5.89	8.76E-07	5.89	0.55
hsa-miR-30a	-12.73	4.36E-15	12.73	0.89
hsa-miR-145	-2.44	1.97E-02	2.44	0.39
hsa-miR-369-3p	-4.12	2.07E-04	4.12	0.54
hsa-miR-32	4.75	3.03E-05	4.75	-0.47
hsa-miR-302a*	3.63	8.48E-04	3.63	-0.50
hsa-miR-20a	7.48	6.54E-09	7.48	-0.75
hsa-miR-154	-9.11	5.51E-11	9.11	0.83
hsa-miR-183	5.41	3.90E-06	5.41	-0.56
hsa-miR-421	6.50	1.34E-07	6.50	-0.55
hsa-miR-183*	3.82	4.91E-04	3.82	-0.39
hsa-miR-331-3p	4.78	2.79E-05	4.78	-0.55
hsa-miR-136	-9.28	3.42E-11	9.28	0.85
hsa-miR-196a	9.91	5.80E-12	9.91	-0.70
hsa-miR-100	-8.65	2.06E-10	8.65	0.73
hsa-miR-499-5p	-8.69	1.80E-10	8.69	0.77
hsa-miR-29c	-6.64	8.57E-08	6.64	0.71
hsa-miR-574-3p	-4.80	2.58E-05	4.80	0.57
hsa-miR-125a-5p	-5.33	5.04E-06	5.33	0.46
hsa-miR-221	4.24	1.44E-04	4.24	-0.60
hsa-miR-139-3p	-5.93	7.81E-07	5.93	0.57
hsa-miR-33a	3.70	7.04E-04	3.70	-0.43
hsa-miR-30e*	-10.02	4.33E-12	10.02	0.71
hsa-miR-611	-2.17	3.61E-02	2.17	0.36
hsa-miR-455-5p	11.98	2.69E-14	11.98	-0.73
hsa-miR-425	2.73	9.59E-03	2.73	-0.35
hsa-miR-30c	-13.68	4.82E-16	13.68	0.85
hsa-miR-34c-5p	9.07	6.09E-11	9.07	-0.68
hsa-miR-93	6.65	8.29E-08	6.65	-0.78
hsa-miR-210	3.49	1.26E-03	3.49	-0.52
hsa-miR-525-3p	-7.09	2.14E-08	7.09	0.53
hsa-miR-19a	5.43	3.69E-06	5.43	-0.55
hsa-miR-487b	-11.22	1.83E-13	11.22	0.87
hsa-miR-661	5.67	1.78E-06	5.67	-0.58
hsa-miR-101	-2.85	7.20E-03	2.85	0.30
hsa-miR-432	-10.62	8.58E-13	10.62	0.81
hsa-miR-135a	-8.44	3.72E-10	8.44	0.65
hsa-miR-31	6.96	3.22E-08	6.96	-0.74
hsa-miR-106b	2.90	6.27E-03	2.90	-0.36
hsa-miR-17*	2.36	2.35E-02	2.36	-0.34
hsa-miR-135b	4.98	1.50E-05	4.98	-0.50
hsa-miR-146b-5p	7.44	7.49E-09	7.44	-0.58
hsa-miR-149	-4.74	3.10E-05	4.74	0.37
hsa-miR-299-5p	-10.17	2.89E-12	10.17	0.81

hsa-miR-193b	6.38	1.91E-07	6.38	-0.62
hsa-miR-18b	4.43	8.05E-05	4.43	-0.43
hsa-miR-765	-3.66	7.90E-04	3.66	0.39
hsa-miR-222	6.34	2.19E-07	6.34	-0.72
hsa-miR-409-3p	-2.55	1.51E-02	2.55	0.46
hsa-let-7a	-7.83	2.31E-09	7.83	0.59
hsa-miR-498	8.11	9.90E-10	8.11	-0.58
hsa-miR-411	-10.03	4.20E-12	10.03	0.85
hsa-miR-16	4.61	4.66E-05	4.61	-0.55
hsa-miR-889	6.69	7.41E-08	6.69	-0.43
hsa-miR-23b	-6.84	4.70E-08	6.84	0.50
hsa-miR-26a	-8.68	1.85E-10	8.68	0.76
hsa-miR-142-3p	2.68	1.08E-02	2.68	-0.34
hsa-miR-206	-3.52	1.15E-03	3.52	0.45
hsa-miR-769-3p	-3.12	3.50E-03	3.12	0.37
hsa-miR-134	-4.53	5.93E-05	4.53	0.60
hsa-miR-548d-5p	-5.88	9.24E-07	5.88	0.46
hsa-miR-658	4.69	3.66E-05	4.69	-0.47
hsa-miR-376a	-5.04	1.25E-05	5.04	0.60
hsa-miR-1228*	6.19	3.49E-07	6.19	-0.61
hsa-miR-655	-6.48	1.42E-07	6.48	0.69
hsa-miR-30e	-1.86	7.04E-02	1.86	0.34
hsa-miR-520h,hsa-miR-520g	5.01	1.36E-05	5.01	-0.51
hsa-miR-187	3.56	1.04E-03	3.56	-0.31
hsa-miR-155	6.23	3.06E-07	6.23	-0.59
hsa-miR-493	-5.17	8.35E-06	5.17	0.64
hsa-miR-378*	-7.82	2.36E-09	7.82	0.74
hsa-miR-450a	7.98	1.46E-09	7.98	-0.56
hsa-miR-101*	-5.09	1.07E-05	5.09	0.56
hsa-miR-328	-6.22	3.12E-07	6.22	0.63
hsa-miR-132	6.28	2.64E-07	6.28	-0.42
hsa-miR-99a	-10.10	3.53E-12	10.10	0.78
hsa-miR-23a	3.28	2.25E-03	3.28	-0.38
hsa-miR-548d-3p	3.52	1.15E-03	3.52	-0.37
hsa-miR-181b	6.09	4.67E-07	6.09	-0.61
hsa-miR-503	16.40	1.46E-18	16.40	-0.75
hsa-miR-941	5.91	8.24E-07	5.91	-0.47
hsa-miR-18a	7.74	2.98E-09	7.74	-0.74
hsa-miR-453	12.16	1.72E-14	12.16	-0.72
hsa-miR-365	5.68	1.68E-06	5.68	-0.32
hsa-miR-339-5p	6.01	6.01E-07	6.01	-0.53
hsa-miR-196b	7.42	7.90E-09	7.42	-0.63
hsa-miR-133a	-6.46	1.51E-07	6.46	0.68
hsa-let-7i	9.20	4.29E-11	9.20	-0.63
hsa-miR-454	3.07	3.98E-03	3.07	-0.36
hsa-miR-1	-6.27	2.67E-07	6.27	0.69
hsa-miR-550*	5.55	2.59E-06	5.55	-0.55
hsa-miR-488*	1.88	6.80E-02	1.88	-0.35
hsa-miR-363	-4.71	3.40E-05	4.71	0.45
hsa-miR-152	-2.34	2.49E-02	2.34	0.44
hsa-miR-133b	-4.69	3.64E-05	4.69	0.54
hsa-miR-519d	6.10	4.53E-07	6.10	-0.59
hsa-miR-181a*	7.09	2.12E-08	7.09	-0.55
hsa-miR-606	-3.96	3.28E-04	3.96	0.46

hsa-miR-218	-7.67	3.72E-09	7.67	0.74
hsa-miR-7	13.52	6.81E-16	13.52	-0.84
hsa-miR-125b	-9.73	9.72E-12	9.73	0.80
hsa-miR-361-5p	-4.49	6.64E-05	4.49	0.59
hsa-miR-598	-4.29	1.22E-04	4.29	0.43
hsa-miR-494	-2.61	1.31E-02	2.61	0.48
hsa-miR-484	4.13	2.01E-04	4.13	-0.49
hsa-miR-656	-3.01	4.74E-03	3.01	0.46
hsa-miR-25	6.40	1.80E-07	6.40	-0.67
hsa-miR-381	-7.46	7.05E-09	7.46	0.67
hsa-miR-377	-3.60	9.40E-04	3.60	0.48
hsa-miR-130a	7.31	1.09E-08	7.31	-0.64
hsa-miR-142-5p	5.72	1.48E-06	5.72	-0.57
hsa-let-7c	-14.64	5.69E-17	14.64	0.87
hsa-miR-224	3.79	5.31E-04	3.79	-0.52
hsa-miR-106a	7.03	2.59E-08	7.03	-0.81
hsa-miR-543	-2.96	5.36E-03	2.96	0.41
hsa-miR-518e	3.93	3.56E-04	3.93	-0.44
hsa-miR-944	6.33	2.26E-07	6.33	-0.61
hsa-miR-362-3p	8.28	6.04E-10	8.28	-0.51
hsa-miR-708	4.80	2.63E-05	4.80	-0.54
hsa-miR-1244	5.22	7.21E-06	5.22	-0.52
hsa-miR-34c-3p	5.92	8.16E-07	5.92	-0.62
hsa-miR-1246	7.36	9.58E-09	7.36	-0.70
hsa-miR-220c	6.40	1.79E-07	6.40	-0.54
hsa-miR-1247	-4.56	5.40E-05	4.56	0.50
hsa-miR-1307	4.31	1.14E-04	4.31	-0.50
hsa-miR-29a*	-5.72	1.50E-06	5.72	0.52
hsa-miR-377*	-3.67	7.67E-04	3.67	0.48
hsa-miR-1256	-3.84	4.67E-04	3.84	0.43
hsa-miR-25*	7.41	8.08E-09	7.41	-0.58
hsa-miR-149*	3.26	2.42E-03	3.26	-0.37
hsa-miR-628-5p	-6.66	7.97E-08	6.66	0.70
hsa-miR-135b*	5.04	1.26E-05	5.04	-0.51
hsa-miR-1826	5.17	8.25E-06	5.17	-0.41
hsa-miR-424*	11.25	1.69E-13	11.25	-0.69
hsa-miR-17	5.71	1.57E-06	5.71	-0.78
hsa-miR-7-1*	5.48	3.21E-06	5.48	-0.55
hsa-miR-21*	14.78	4.22E-17	14.78	-0.84
hsa-miR-99a*	-4.14	1.94E-04	4.14	0.47
hsa-miR-34b	4.59	4.93E-05	4.59	-0.41
hsa-miR-32*	6.32	2.32E-07	6.32	-0.53
hsa-miR-1183	3.52	1.15E-03	3.52	-0.44
hsa-miR-1265	-5.73	1.47E-06	5.73	0.45
hsa-miR-136*	-7.79	2.58E-09	7.79	0.81
hsa-miR-200b*	-5.36	4.63E-06	5.36	0.50
hsa-miR-16-2*	2.00	5.33E-02	2.00	-0.31
hsa-miR-935	-2.96	5.27E-03	2.96	0.42
hsa-miR-106b*	4.29	1.24E-04	4.29	-0.46
hsa-miR-532-3p	-2.46	1.86E-02	2.46	0.44
hsa-miR-1267	11.43	1.07E-13	11.43	-0.82
hsa-miR-15b*	5.07	1.14E-05	5.07	-0.55
hsa-miR-1268	9.26	3.61E-11	9.26	-0.74
hsa-miR-486-3p	-5.95	7.42E-07	5.95	0.54
hsa-miR-331-5p	5.76	1.31E-06	5.76	-0.53

hsa-miR-921	-12.34	1.12E-14	12.34	0.79
hsa-miR-222*	3.53	1.12E-03	3.53	-0.40
hsa-miR-452*	5.58	2.33E-06	5.58	-0.47
hsa-miR-337-3p	-8.29	5.77E-10	8.29	0.81
hsa-miR-127-5p	-2.01	5.21E-02	2.01	0.33
hsa-let-7i*	4.93	1.73E-05	4.93	-0.42
hsa-miR-30c-2*	-4.81	2.50E-05	4.81	0.56
hsa-miR-29b-2*	-5.98	6.60E-07	5.98	0.57
hsa-miR-1271	-6.87	4.27E-08	6.87	0.53
hsa-miR-337-5p	-7.20	1.55E-08	7.20	0.64
hsa-miR-1272	-8.04	1.21E-09	8.04	0.74
hsa-miR-455-3p	11.20	1.91E-13	11.20	-0.82
hsa-miR-1308	5.12	9.81E-06	5.12	-0.63
hsa-miR-186*	3.58	9.85E-04	3.58	-0.49
hsa-miR-145*	-5.80	1.17E-06	5.80	0.69
hsa-miR-663b	11.33	1.38E-13	11.33	-0.75
hsa-miR-376a*	-4.79	2.71E-05	4.79	0.64
hsa-miR-887	-4.89	1.97E-05	4.89	0.48
hsa-miR-1226*	7.23	1.39E-08	7.23	-0.63
hsa-miR-124	3.62	8.78E-04	3.62	-0.48
hsa-miR-140-3p	-6.21	3.23E-07	6.21	0.61
hsa-miR-300	-4.06	2.44E-04	4.06	0.40
hsa-miR-411*	-4.50	6.46E-05	4.50	0.53
hsa-miR-379*	-5.07	1.15E-05	5.07	0.62
hsa-miR-125b-2*	-6.95	3.31E-08	6.95	0.66
hsa-miR-128	-3.92	3.70E-04	3.92	0.46
hsa-miR-885-5p	-9.28	3.42E-11	9.28	0.76
hsa-miR-1204	8.63	2.17E-10	8.63	-0.66
hsa-miR-29c*	-10.58	9.62E-13	10.58	0.83
hsa-miR-423-5p	3.79	5.35E-04	3.79	-0.42
hsa-miR-938	3.36	1.83E-03	3.36	-0.42
hsa-miR-24-1*	-3.69	7.11E-04	3.69	0.39
hsa-miR-31*	6.96	3.22E-08	6.96	-0.72
hsa-miR-1290	8.32	5.40E-10	8.32	-0.76
hsa-miR-933	-6.95	3.28E-08	6.95	0.62
hsa-miR-1300	5.01	1.38E-05	5.01	-0.46
hsa-miR-450b-5p	5.32	5.27E-06	5.32	-0.35

Supplementary Table 2. List of the 309 miRNAs in the grey module.

miRNA	stat	pval	GS	MMgrey
hsa-miR-515-5p	3.38	1.72E-03	3.38	0.34
hsa-miR-383	-3.30	2.15E-03	3.30	-0.32
hsa-miR-324-5p	2.72	1.00E-02	2.72	0.29
hsa-miR-599	2.42	2.06E-02	2.42	0.20
hsa-miR-555	-1.68	1.02E-01	1.68	-0.20
hsa-miR-200c	3.02	4.58E-03	3.02	0.42
hsa-miR-103	1.92	6.30E-02	1.92	0.09
hsa-miR-29a	-3.34	1.90E-03	3.34	-0.49
hsa-miR-452	2.02	5.03E-02	2.02	0.26
hsa-miR-671-3p	1.74	9.07E-02	1.74	0.08
hsa-miR-520f	2.24	3.11E-02	2.24	0.19
hsa-miR-205	0.66	5.12E-01	0.66	-0.03
hsa-miR-579	2.83	7.48E-03	2.83	0.10
hsa-miR-302a	-1.04	3.05E-01	1.04	0.04
hsa-miR-194	-2.31	2.65E-02	2.31	-0.24
hsa-miR-517a,hsa-miR-517b	0.71	4.80E-01	0.71	0.26
hsa-miR-208a	0.07	9.45E-01	0.07	0.06
hsa-miR-622	3.11	3.62E-03	3.11	0.51
hsa-miR-185	1.70	9.82E-02	1.70	-0.35
hsa-miR-200a*	-0.76	4.52E-01	0.76	-0.18
hsa-miR-629*	0.27	7.89E-01	0.27	-0.10
hsa-let-7f	-0.08	9.35E-01	0.08	-0.49
hsa-miR-367	0.04	9.65E-01	0.04	0.05
hsa-miR-296-5p	-0.47	6.39E-01	0.47	-0.22
hsa-miR-624*	0.77	4.48E-01	0.77	-0.01
hsa-miR-373*	1.19	2.43E-01	1.19	0.00
hsa-miR-584	1.50	1.41E-01	1.50	0.12
hsa-miR-639	0.64	5.25E-01	0.64	0.25
hsa-miR-576-5p	0.34	7.33E-01	0.34	0.00
hsa-miR-637	1.34	1.88E-01	1.34	0.23
hsa-miR-1296	-1.45	1.56E-01	1.45	-0.17
hsa-miR-216a	-1.19	2.41E-01	1.19	-0.18
hsa-miR-512-3p	2.88	6.64E-03	2.88	0.06
hsa-miR-211	-3.71	6.71E-04	3.71	-0.32
hsa-miR-423-3p	-0.98	3.36E-01	0.98	-0.31
hsa-miR-633	2.86	6.96E-03	2.86	0.37
hsa-miR-496	1.68	1.02E-01	1.68	0.10
hsa-miR-564	-3.36	1.82E-03	3.36	-0.49
hsa-miR-320d,hsa-miR-320b,hsa-miR-320a,hsa-miR-320c	-1.39	1.72E-01	1.39	-0.03
hsa-miR-675	-1.00	3.24E-01	1.00	-0.22
hsa-miR-563	2.00	5.25E-02	2.00	0.09
hsa-miR-198	3.40	1.61E-03	3.40	0.33
hsa-miR-575	0.33	7.46E-01	0.33	-0.11
hsa-miR-15a	2.05	4.75E-02	2.05	0.22
hsa-miR-589*	0.59	5.58E-01	0.59	-0.09
hsa-miR-192	-1.84	7.41E-02	1.84	-0.08
hsa-miR-513c	-1.16	2.55E-01	1.16	-0.17
hsa-miR-380	-0.40	6.90E-01	0.40	0.18
hsa-miR-449a	2.16	3.72E-02	2.16	0.29
hsa-miR-720	1.81	7.87E-02	1.81	0.38

hsa-miR-373	-0.31	7.62E-01	0.31	-0.03
hsa-miR-645	0.53	5.99E-01	0.53	-0.21
hsa-miR-217	-0.24	8.15E-01	0.24	0.05
hsa-miR-572	-0.40	6.88E-01	0.40	0.20
hsa-miR-608	2.93	5.78E-03	2.93	0.19
hsa-miR-363*	-2.83	7.41E-03	2.83	-0.35
hsa-miR-586	-1.81	7.88E-02	1.81	-0.38
hsa-miR-219-5p	1.65	1.07E-01	1.65	0.08
hsa-miR-650	3.98	3.11E-04	3.98	0.34
hsa-miR-122	-2.63	1.23E-02	2.63	-0.09
hsa-let-7d	0.42	6.77E-01	0.42	-0.12
hsa-miR-10a	1.05	2.98E-01	1.05	-0.19
hsa-miR-20b	-3.86	4.36E-04	3.86	-0.35
hsa-miR-644	1.81	7.80E-02	1.81	0.32
hsa-miR-662	0.16	8.74E-01	0.16	0.04
hsa-miR-451	-2.59	1.36E-02	2.59	-0.26
hsa-miR-1251	-1.21	2.32E-01	1.21	-0.04
hsa-miR-556-5p	-0.48	6.35E-01	0.48	-0.07
hsa-miR-1537	3.42	1.52E-03	3.42	0.40
hsa-miR-220a	-1.81	7.86E-02	1.81	-0.11
hsa-miR-1231	1.12	2.70E-01	1.12	0.09
hsa-miR-612	3.60	9.30E-04	3.60	0.34
hsa-miR-181d	-1.14	2.60E-01	1.14	-0.22
hsa-miR-126	-3.76	5.89E-04	3.76	-0.64
hsa-miR-184	0.87	3.89E-01	0.87	0.10
hsa-let-7b*	3.15	3.23E-03	3.15	0.17
hsa-miR-566	2.65	1.18E-02	2.65	0.33
hsa-miR-526b*	2.59	1.36E-02	2.59	0.29
hsa-miR-215	-0.11	9.15E-01	0.11	-0.17
hsa-miR-580	-0.89	3.81E-01	0.89	-0.07
hsa-miR-182	1.90	6.52E-02	1.90	-0.25
hsa-miR-616*	-1.48	1.47E-01	1.48	-0.18
hsa-miR-22	3.95	3.39E-04	3.95	0.29
hsa-miR-432*	-2.19	3.49E-02	2.19	-0.28
hsa-miR-200a	0.45	6.55E-01	0.45	0.13
hsa-miR-604	1.62	1.13E-01	1.62	0.15
hsa-miR-429	-0.62	5.40E-01	0.62	-0.23
hsa-miR-623	1.54	1.32E-01	1.54	0.22
hsa-miR-570	-0.24	8.14E-01	0.24	0.01
hsa-miR-522	3.61	9.11E-04	3.61	0.30
hsa-miR-885-3p	0.20	8.41E-01	0.20	0.05
hsa-miR-630	-0.40	6.93E-01	0.40	0.03
hsa-miR-552	1.34	1.87E-01	1.34	-0.04
hsa-miR-588	1.83	7.50E-02	1.83	0.14
hsa-miR-767-3p	0.69	4.93E-01	0.69	0.22
hsa-miR-326	0.62	5.36E-01	0.62	0.41
hsa-miR-422a	1.21	2.35E-01	1.21	0.30
hsa-miR-376b	-0.93	3.59E-01	0.93	-0.02
hsa-miR-519c-3p	1.65	1.07E-01	1.65	0.10
hsa-miR-873	0.80	4.27E-01	0.80	0.17
hsa-miR-199a-5p	-0.36	7.19E-01	0.36	-0.01
hsa-miR-335	-1.11	2.73E-01	1.11	-0.32
hsa-miR-520a-5p	0.07	9.47E-01	0.07	-0.04
hsa-miR-659	0.11	9.09E-01	0.11	-0.35
hsa-miR-380*	-0.33	7.44E-01	0.33	0.26

hsa-miR-519e*	1.77	8.54E-02	1.77	0.04
hsa-miR-631	2.26	2.95E-02	2.26	0.18
hsa-miR-520c-3p,hsa-miR-520f	1.53	1.36E-01	1.53	0.12
hsa-miR-296-3p	-2.56	1.48E-02	2.56	-0.33
hsa-miR-141	1.79	8.20E-02	1.79	0.28
hsa-miR-518e*,hsa-miR-519a*,hsa-miR-519b-5p,hsa-miR-519c-5p,hsa-miR-522*,hsa-miR-523*	3.00	4.79E-03	3.00	0.25
hsa-miR-510	-0.34	7.38E-01	0.34	-0.20
hsa-miR-638	0.27	7.91E-01	0.27	0.15
hsa-miR-597	-1.56	1.26E-01	1.56	-0.27
hsa-miR-485-3p	-0.84	4.07E-01	0.84	-0.14
hsa-miR-519a	3.24	2.55E-03	3.24	0.23
hsa-miR-29b	3.25	2.46E-03	3.25	0.35
hsa-miR-518f*,hsa-miR-518d-5p,hsa-miR-520c-5p,hsa-miR-526a	0.15	8.82E-01	0.15	0.08
hsa-miR-24	-0.41	6.81E-01	0.41	-0.04
hsa-miR-521	-0.11	9.16E-01	0.11	0.17
hsa-miR-760	0.77	4.44E-01	0.77	-0.07
hsa-miR-548c-3p	0.10	9.24E-01	0.10	-0.06
hsa-miR-627	-0.63	5.30E-01	0.63	0.27
hsa-miR-583	-1.45	1.57E-01	1.45	-0.30
hsa-miR-448	0.99	3.31E-01	0.99	0.00
hsa-miR-374a	2.86	6.94E-03	2.86	-0.22
hsa-miR-1185	-0.61	5.48E-01	0.61	0.04
hsa-miR-520e	1.32	1.94E-01	1.32	0.30
hsa-miR-876-3p	1.38	1.77E-01	1.38	0.31
hsa-miR-512-5p	2.51	1.64E-02	2.51	0.39
hsa-miR-544	2.06	4.60E-02	2.06	-0.09
hsa-miR-490-3p	-1.50	1.43E-01	1.50	-0.23
hsa-miR-524-5p	1.14	2.62E-01	1.14	-0.13
hsa-miR-200b	-0.29	7.72E-01	0.29	-0.04
hsa-miR-27a	2.72	9.86E-03	2.72	-0.20
hsa-miR-632	-1.81	7.85E-02	1.81	0.22
hsa-miR-648	-0.41	6.82E-01	0.41	-0.18
hsa-miR-520g	2.47	1.83E-02	2.47	0.20
hsa-miR-509-3p	-3.47	1.32E-03	3.47	-0.24
hsa-miR-636	2.40	2.13E-02	2.40	0.22
hsa-miR-559	1.77	8.53E-02	1.77	0.31
hsa-miR-591	-1.60	1.18E-01	1.60	-0.53
hsa-miR-605	1.02	3.14E-01	1.02	0.16
hsa-miR-126*	-0.04	9.65E-01	0.04	-0.23
hsa-miR-27b	-0.78	4.38E-01	0.78	-0.40
hsa-miR-324-3p	-3.22	2.66E-03	3.22	-0.20
hsa-miR-558	0.82	4.16E-01	0.82	0.39
hsa-miR-587	0.82	4.20E-01	0.82	0.08
hsa-miR-1468	-3.56	1.05E-03	3.56	-0.31
hsa-miR-770-5p	-0.31	7.57E-01	0.31	0.09
hsa-let-7e	-1.09	2.85E-01	1.09	-0.31
hsa-miR-514	-3.38	1.71E-03	3.38	-0.32

hsa-miR-146a	1.84	7.33E-02	1.84	0.26
hsa-miR-504	-0.23	8.19E-01	0.23	0.25
hsa-miR-621	-1.10	2.78E-01	1.10	-0.17
hsa-miR-619	-0.87	3.92E-01	0.87	-0.06
hsa-miR-590-5p	0.20	8.39E-01	0.20	-0.13
hsa-miR-96	1.51	1.41E-01	1.51	-0.37
hsa-miR-635	1.22	2.32E-01	1.22	0.18
hsa-miR-562	2.82	7.66E-03	2.82	0.17
hsa-miR-548e	1.12	2.72E-01	1.12	0.03
hsa-miR-548a-5p	-0.33	7.41E-01	0.33	0.12
hsa-miR-548k	1.44	1.58E-01	1.44	0.28
hsa-miR-548l	-2.55	1.49E-02	2.55	-0.18
hsa-miR-541*	-0.29	7.70E-01	0.29	0.18
hsa-miR-520d-3p	0.70	4.91E-01	0.70	0.09
hsa-miR-216b	0.76	4.54E-01	0.76	-0.31
hsa-miR-499-3p	-2.15	3.82E-02	2.15	-0.23
hsa-miR-483-5p	0.80	4.28E-01	0.80	-0.07
hsa-miR-1179	-0.27	7.86E-01	0.27	0.05
hsa-miR-129-3p	-1.06	2.95E-01	1.06	-0.01
hsa-miR-129*	0.94	3.53E-01	0.94	0.21
hsa-miR-148b*	2.20	3.42E-02	2.20	0.19
hsa-miR-96*	-0.09	9.26E-01	0.09	-0.14
hsa-miR-936	0.32	7.53E-01	0.32	0.16
hsa-miR-199a-3p,hsa-miR-199b-3p	-0.37	7.11E-01	0.37	0.10
hsa-miR-181a-2*	1.52	1.38E-01	1.52	0.13
hsa-miR-1248	-2.18	3.59E-02	2.18	-0.53
hsa-miR-125b-1*	-1.89	6.65E-02	1.89	0.06
hsa-miR-1250	3.00	4.76E-03	3.00	0.23
hsa-miR-371-5p	-0.93	3.60E-01	0.93	-0.30
hsa-miR-20a*	-2.54	1.53E-02	2.54	-0.13
hsa-miR-20b*	-1.58	1.22E-01	1.58	-0.35
hsa-miR-302d*	-0.05	9.61E-01	0.05	-0.09
hsa-miR-219-2-3p	-2.47	1.84E-02	2.47	-0.35
hsa-miR-27b*	-2.43	2.01E-02	2.43	-0.09
hsa-miR-219-1-3p	1.70	9.84E-02	1.70	0.25
hsa-miR-1201	2.44	1.96E-02	2.44	0.29
hsa-miR-671-5p	-1.13	2.64E-01	1.13	-0.08
hsa-miR-1255a	0.98	3.36E-01	0.98	0.20
hsa-miR-92b*	2.45	1.93E-02	2.45	0.17
hsa-miR-27a*	1.30	2.01E-01	1.30	-0.14
hsa-miR-185*	1.50	1.43E-01	1.50	0.19
hsa-miR-92a-1*	-0.20	8.43E-01	0.20	0.01
hsa-miR-616	-0.84	4.09E-01	0.84	-0.25
hsa-miR-550	-1.53	1.33E-01	1.53	-0.41
hsa-miR-1258	0.64	5.23E-01	0.64	-0.12
hsa-miR-22*	2.60	1.32E-02	2.60	0.44
hsa-miR-19b-1*	0.30	7.65E-01	0.30	0.25
hsa-miR-19a*	1.57	1.26E-01	1.57	0.16
hsa-miR-1259	-0.73	4.71E-01	0.73	-0.21
hsa-miR-556-3p	-2.20	3.41E-02	2.20	-0.24
hsa-miR-937	-1.58	1.22E-01	1.58	-0.32
hsa-miR-548n	2.40	2.14E-02	2.40	0.23
hsa-miR-497*	-0.48	6.31E-01	0.48	0.10
hsa-miR-7-2*	-2.32	2.61E-02	2.32	-0.27

hsa-miR-708*	3.14	3.30E-03	3.14	0.43
hsa-miR-100*	1.12	2.70E-01	1.12	-0.04
hsa-miR-1264	-1.21	2.34E-01	1.21	-0.08
hsa-miR-15a*	3.14	3.35E-03	3.14	0.44
hsa-miR-301b	1.52	1.37E-01	1.52	0.19
hsa-miR-33b*	1.20	2.38E-01	1.20	0.23
hsa-miR-141*	0.08	9.35E-01	0.08	0.04
hsa-miR-548o	0.66	5.15E-01	0.66	0.01
hsa-miR-1324	0.81	4.24E-01	0.81	0.27
hsa-miR-1203	-0.58	5.67E-01	0.58	-0.15
hsa-miR-1181	1.63	1.11E-01	1.63	0.35
hsa-miR-26a-1*	-1.41	1.66E-01	1.41	0.14
hsa-miR-1266	-0.67	5.07E-01	0.67	-0.17
hsa-miR-146a*	2.94	5.63E-03	2.94	0.31
hsa-miR-1236	0.56	5.81E-01	0.56	0.21
hsa-miR-26b*	-2.40	2.18E-02	2.40	-0.32
hsa-miR-886-3p	0.02	9.87E-01	0.02	0.04
hsa-miR-1255b	-0.53	5.96E-01	0.53	-0.23
hsa-miR-196a*	0.98	3.32E-01	0.98	0.03
hsa-miR-193b*	1.75	8.78E-02	1.75	0.09
hsa-miR-886-5p	2.23	3.20E-02	2.23	0.05
hsa-miR-200c*	0.55	5.84E-01	0.55	-0.01
hsa-miR-1227	0.92	3.65E-01	0.92	-0.01
hsa-miR-223*	2.81	7.78E-03	2.81	0.10
hsa-miR-124*	0.76	4.50E-01	0.76	0.01
hsa-miR-151-3p	1.31	1.99E-01	1.31	0.31
hsa-let-7a*	0.63	5.30E-01	0.63	-0.31
hsa-let-7f-2*	-1.99	5.37E-02	1.99	-0.41
hsa-miR-188-3p	0.17	8.65E-01	0.17	-0.13
hsa-miR-155*	3.93	3.55E-04	3.93	0.34
hsa-miR-106a*	-1.16	2.55E-01	1.16	-0.08
hsa-miR-192*	0.00	1.00E+00	0.00	0.10
hsa-miR-874	-1.86	7.08E-02	1.86	0.04
hsa-miR-1269	2.53	1.59E-02	2.53	-0.03
hsa-miR-1270	-8.15	8.91E-10	8.15	-0.67
hsa-miR-30c-1*	-3.56	1.04E-03	3.56	-0.41
hsa-miR-30b*	0.48	6.32E-01	0.48	-0.06
hsa-let-7g*	0.41	6.84E-01	0.41	-0.01
hsa-miR-374b*	3.61	9.13E-04	3.61	0.15
hsa-miR-374a*	0.03	9.74E-01	0.03	0.01
hsa-miR-1238	1.70	9.77E-02	1.70	0.41
hsa-miR-30d*	0.11	9.17E-01	0.11	-0.14
hsa-miR-518f	2.21	3.34E-02	2.21	0.38
hsa-miR-551b*	2.20	3.43E-02	2.20	0.29
hsa-miR-625*	-0.89	3.79E-01	0.89	-0.18
hsa-miR-888*	2.02	5.12E-02	2.02	0.19
hsa-miR-1182	3.25	2.46E-03	3.25	0.35
hsa-miR-922	-5.15	8.90E-06	5.15	-0.43
hsa-miR-138-1*	0.58	5.64E-01	0.58	0.39
hsa-miR-144*	-3.55	1.06E-03	3.55	-0.42
hsa-miR-505*	0.08	9.39E-01	0.08	-0.11
hsa-miR-1273	0.03	9.77E-01	0.03	-0.06
hsa-miR-23a*	2.00	5.28E-02	2.00	-0.02
hsa-miR-92a-2*	1.99	5.42E-02	1.99	0.10
hsa-miR-923	-0.44	6.64E-01	0.44	-0.15

hsa-miR-1224-5p	0.12	9.09E-01	0.12	0.03
hsa-miR-1225-5p	0.66	5.16E-01	0.66	0.15
hsa-miR-500	-1.57	1.26E-01	1.57	-0.09
hsa-miR-590-3p	2.13	3.98E-02	2.13	0.09
hsa-miR-888	-3.79	5.36E-04	3.79	-0.50
hsa-miR-509-3-5p	-2.33	2.54E-02	2.33	-0.23
hsa-let-7c*	-2.78	8.48E-03	2.78	-0.35
hsa-miR-548p	-5.82	1.11E-06	5.82	-0.66
hsa-miR-1197	1.18	2.45E-01	1.18	0.16
hsa-miR-1278	0.60	5.51E-01	0.60	0.07
hsa-miR-135a*	-0.15	8.80E-01	0.15	0.23
hsa-miR-654-3p	-0.93	3.58E-01	0.93	0.31
hsa-miR-1228	2.06	4.67E-02	2.06	0.18
hsa-miR-1226	4.08	2.32E-04	4.08	0.24
hsa-miR-1207-3p	-0.59	5.61E-01	0.59	0.05
hsa-miR-1280	-0.11	9.14E-01	0.11	0.41
hsa-miR-1237	2.71	1.02E-02	2.71	0.16
hsa-miR-1281	1.45	1.55E-01	1.45	0.19
hsa-miR-1234	-3.19	2.89E-03	3.19	-0.05
hsa-miR-1282	-2.96	5.30E-03	2.96	-0.21
hsa-miR-330-5p	0.95	3.48E-01	0.95	0.12
hsa-miR-942	-0.14	8.88E-01	0.14	0.12
hsa-miR-589	2.27	2.93E-02	2.27	0.31
hsa-miR-574-5p	-0.87	3.91E-01	0.87	-0.11
hsa-miR-891b	-0.90	3.76E-01	0.90	-0.02
hsa-miR-24-2*	1.56	1.28E-01	1.56	0.10
hsa-miR-744	-0.59	5.57E-01	0.59	0.02
hsa-miR-1288	-2.22	3.24E-02	2.22	-0.50
hsa-miR-1207-5p	1.36	1.81E-01	1.36	0.03
hsa-miR-1291	0.06	9.56E-01	0.06	-0.05
hsa-miR-1292	0.15	8.78E-01	0.15	0.18
hsa-miR-193a-5p	0.32	7.49E-01	0.32	0.21
hsa-miR-1293	3.76	5.80E-04	3.76	0.25
hsa-miR-23b*	-2.71	1.01E-02	2.71	-0.04
hsa-miR-629	-0.56	5.77E-01	0.56	-0.12
hsa-miR-541	1.40	1.71E-01	1.40	0.15
hsa-miR-1294	-1.25	2.18E-01	1.25	-0.51
hsa-miR-1295	1.88	6.77E-02	1.88	0.40
hsa-miR-513b	-0.20	8.40E-01	0.20	-0.29
hsa-miR-130a*	-0.66	5.16E-01	0.66	-0.42
hsa-miR-202*	-0.62	5.37E-01	0.62	-0.07
hsa-miR-488	-0.66	5.15E-01	0.66	-0.13
hsa-miR-1301	1.77	8.49E-02	1.77	0.19
hsa-miR-1178	1.57	1.26E-01	1.57	0.22
hsa-miR-1302	0.31	7.62E-01	0.31	0.07
hsa-miR-1303	2.69	1.05E-02	2.69	0.44
hsa-miR-1304	-1.21	2.34E-01	1.21	-0.09
hsa-miR-335*	-0.09	9.28E-01	0.09	0.04
